# Normal formation of a vertebrate body plan and loss of tissue maintenance in the absence of *ezh2*

**DOI:** 10.1038/srep24658

**Published:** 2016-05-05

**Authors:** Bilge San, Naomi D. Chrispijn, Nadine Wittkopp, Simon J. van Heeringen, Anne K. Lagendijk, Marco Aben, Jeroen Bakkers, René F. Ketting, Leonie M. Kamminga

**Affiliations:** 1 Radboud University Medical Center, Radboud Institute for Molecular Life Sciences, Nijmegen, The Netherlands; 2 Radboud University, Faculty of Science, Department of Molecular Biology, Radboud Institute for Molecular Life Sciences, Nijmegen, The Netherlands; 3 Hubrecht Institute, University Medical Centre Utrecht, Utrecht, The Netherlands; 4 Institute of Molecular Biology, Mainz, Germany; 5 Radboud University, Faculty of Science, Department of Molecular Developmental Biology, Radboud Institute for Molecular Life Sciences, Nijmegen, The Netherlands; 6 Medical Physiology, University Medical Centre Utrecht, Utrecht, The Netherlands

## Abstract

Polycomb group (PcG) proteins are transcriptional repressors of numerous genes, many of which regulate cell cycle progression or developmental processes. We used zebrafish to study Enhancer of zeste homolog 2 (Ezh2), the PcG protein responsible for placing the transcriptional repressive H3K27me3 mark. We identified a nonsense mutant of *ezh2* and generated maternal zygotic (MZ) *ezh2* mutant embryos. In contrast to knockout mice for PcG proteins, *MZezh2* mutant embryos gastrulate seemingly normal, but die around 2 days post fertilization displaying pleiotropic phenotypes. Expression analyses indicated that genes important for early development are not turned off properly, revealing a regulatory role for Ezh2 during zygotic gene expression. In addition, we suggest that Ezh2 regulates maternal mRNA loading of zygotes. Analyses of tissues arising later in development, such as heart, liver, and pancreas, indicated that Ezh2 is required for maintenance of differentiated cell fates. Our data imply that the primary role of Ezh2 is to maintain tissues after tissue specification. Furthermore, our work indicates that Ezh2 is essential to sustain tissue integrity and to set up proper maternal mRNA contribution, and presents a novel and powerful tool to study how PcG proteins contribute to early vertebrate development.

Early development of multi-cellular organisms is a highly dynamic process requiring an exquisite and tight control over establishment and maintenance of cellular identity. Deregulation of these processes can lead to malformations or disease. Hence, a proper understanding of both cellular differentiation and maintenance of cell fate is relevant in many different settings.

To enable proper cellular specification, expression profiles have to become spatially and temporally restricted during development. Because every cell in theory has the same DNA content gene expression has to be determined at a higher order of regulation. This is in part achieved by chromatin: the complex of DNA wrapped around an octamer of histones plus associated proteins. The histone-octamer contains histones H2A, H2B, H3, and H4, which can be post-translationally modified^1^. In addition, DNA itself can be modified by methylation^2^. The combination of modifications, sometimes also referred to as the epigenome, is thought to determine the accessibility and transcriptional activity of DNA.

One of the protein complexes affecting chromatin modifications is the well-conserved Polycomb group (PcG) complex that was first identified in *Drosophila*. PcG proteins repress gene expression by depositing repressive histone marks, H3K27me3 and H2AK119Ub^3^. Well-known targets of PcG proteins are *Hox* genes^4^. Pioneering work established that PcG proteins are essential for proper patterning during early embryogenesis. In addition, it is proposed that PcG proteins are essential to balance pluripotency and differentiation potential of stem cells^5^,^6^,^7^,^8^. Besides a role in early embryogenesis, PcG proteins are important for tissue-specific development^9^,^10^,^11^,^12^.

PcG proteins are basically found in two complexes, Polycomb Repressive Complex 1 (PRC1) and PRC2. PRC2 contains Enhancer of Zeste Homolog 2 or 1 (EZH2/EZH1), Embryonic Ectoderm Development (EED), and Suppressor of Zeste 12 (SUZ12). In the canonical Polycomb pathway PRC2 is recruited to chromatin before PRC1. EZH2 has a catalytically active SET domain that places the repressive H3K27me3 mark. EZH1 also has methyltransferase activity, although less than EZH2^13^, and is postulated to complement the function of EZH2^14^. In addition, EZH2 is thought to act during proliferation, whereas EZH1 operates more in differentiated cells^15^. Following H3K27 tri-methylation, PRC1 is recruited, allowing the PRC1 component RING1 to ubiquitylate lysine 119 of histone H2A, stabilizing the repressive mark^3^. However, recent studies implicate that PRC1 is also active in the absence of PRC2^16^. In addition, it was shown that PRC1 can promote H3K27 methylation via a positive feedback loop^17^.

Most PcG mouse mutants display pre-gastrulation embryonic lethality^5^,^18^,^19^. In mice, both homologs of RING1, Ring1 and Rnf2, are essential for development of primordial germ cells. During oogenesis Ring1 and Rnf2 serve redundant transcriptional functions, which are essential for proper zygotic genome activation (ZGA). Mutant embryos fail to activate gene transcription and loss of *Ring1* and *Rnf2* has an effect on development-associated genes^20^,^21^.

Although it is clear from published work that PcG proteins are involved in conserved processes that are essential for organismal functioning, many critical questions remain unanswered. For instance, it is not known what their role is during early development of a vertebrate system, a question that can be well addressed in zebrafish. PcG proteins are conserved in zebrafish as well as their accompanying epigenetic marks. Before ZGA, which starts around mid-blastula transition (MBT, 3.3 hours post fertilization) and is accompanied by degradation of maternal transcripts^22^,^23^,^24^,^25^, levels of H3K4me3 (a mark associated with active gene transcription) and H3K27me3 are low. From MBT onwards, the number of genes harboring H3K4me3 increases, which is followed by an increase of RNA Polymerase II occupancy. At the same time the number of genes marked with H3K27me3 slowly increases, suggesting a balance between gene activation and gene repression^25^,^26^,^27^. This also implies that H3K4me3 and H3K27me3 are important during early embryonic development, presumably for cell fate specification or maintenance. A hint for this comes from *rnf2* mutant zebrafish embryos that die around 4–5 days post fertilization (dpf), a time at which organogenesis is normally completed, displaying defects in pectoral fin development^28^.

In this study we generated maternal zygotic mutants for *ezh2* to determine the role of Ezh2 during embryonic development. This unique model system makes it possible to obtain detailed information about the function of Ezh2 during early development. Our data show that Ezh2 is dispensable for gastrulation and tissue specification in zebrafish, despite major overall changes in gene expression, a finding that contrasts phenotypes observed in mice. Furthermore, our data indicate that Ezh2 is required for tissue maintenance in at least three different organs.

## Results

### Ezh2 is conserved in zebrafish

**Figure 1:**
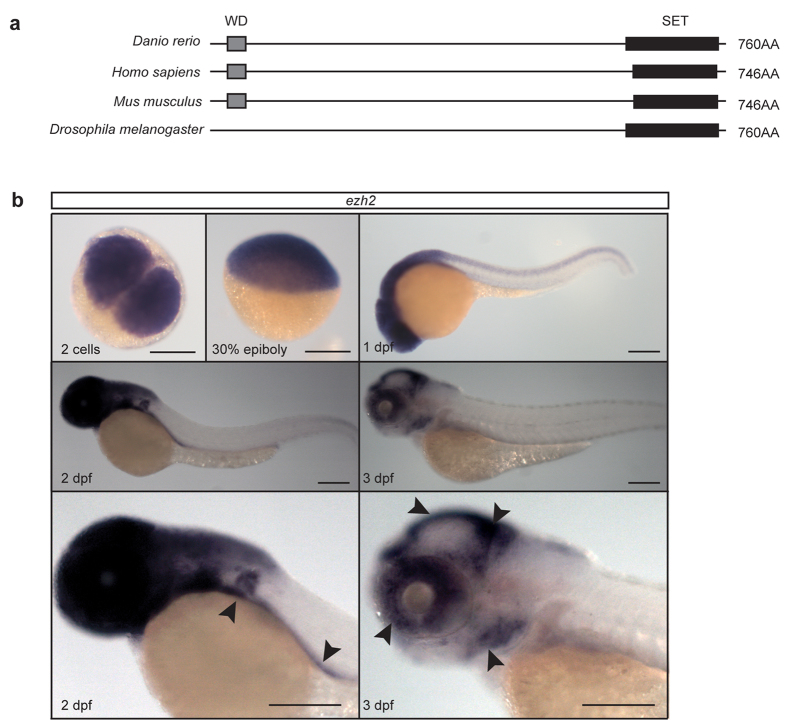
The Polycomb group protein Ezh2 is conserved in zebrafish and *ezh2* mRNA is maternally provided in zebrafish embryos. (**a)** Schematic representation of Ezh2 orthologs in zebrafish, human, mouse, and *Drosophila*. Detailed alignments (Supplementary Fig. S1) show high conservation between the different species. This is 85% and 86% between zebrafish and human and mouse, respectively. Black boxes indicate the location of the SET domain. Grey boxes indicate the location of the WD domain. (**b**) *In situ* hybridization for *ezh2* at 2 cells, 30% epiboly, 1, 2, and 3 dpf. *ezh2* mRNA is maternally provided and at 2 and 3 dpf it is expressed in the pectoral fins, gut, tectum, eye, mid-hindbrain region, and the branchial arches (arrow heads). Scale bar is 200 μm.

The Polycomb group protein Ezh2 is conserved between many species (Fig. 1a and Supplementary Fig. S1). In vertebrates, Ezh2 has a WD repeat domain at the N-terminus, which is implicated in binding Eed and Suz12 (Fig. 1a). In addition, the protein contains a SET domain at the C-terminus, which has histone methyltransferase activity. In contrast to the WD repeat domain, the SET domain is also present in invertebrate species.

When analyzing the mRNA expression profile of *ezh2* in zebrafish we found that *ezh2* mRNA is maternally loaded into the embryo, as we can detect it already at the two-cell stage (Fig. 1b), however Ezh2 protein does not seem to be maternally provided and is only visible after zygotic genome activation (Supplementary Fig. S2). During further early stages of development *ezh2* mRNA is expressed ubiquitously, but becomes more restricted later during development. At 3 dpf *ezh2* expression is restricted to the tectum, mid-hindbrain region, eyes, branchial arches, and gut (Fig. 1b).

### Generation of maternal zygotic ezh2 mutants

**Figure 2:**
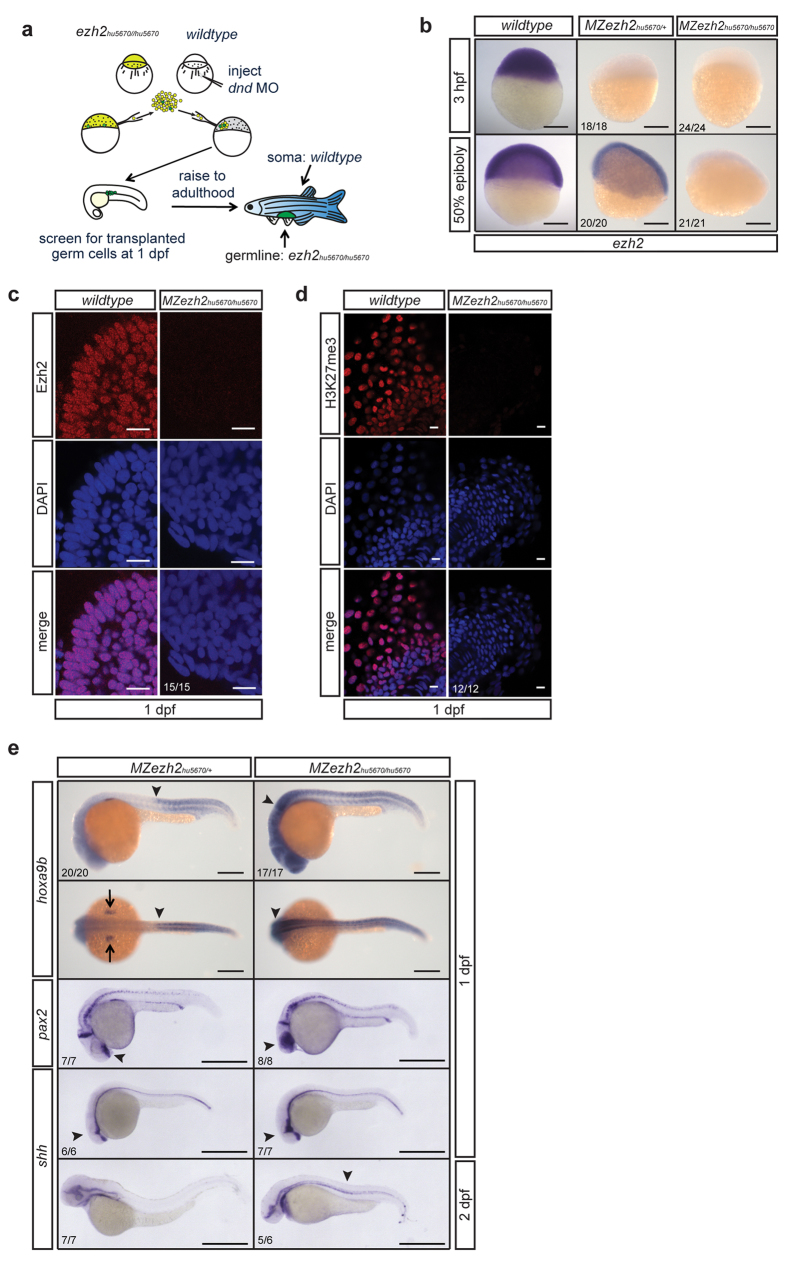
Maternal zygotic *ezh2* mutant embryos lack Ezh2 and H3K27me3, and show aberrant *hox, pax,* and *shh* gene expression. (**a**) Schematic representation of germline transplantation at sphere stage to obtain germline mutant zebrafish. The progeny are maternal zygotic *ezh2* mutant embryos (*MZezh2*^*hu5670*/*hu5670*^). (**b**) *In situ* hybridization for *ezh2* mRNA shows maternal contribution of *ezh2* as well as zygotic expression in wildtype embryos. Maternal contribution of *ezh2* is lost (3 hpf) in *MZezh2*^*hu5670*/+^ and *MZezh2*^*hu5670*/*hu5670*^ embryos. Zygotic *ezh2* expression (30% epiboly) is also lost in *MZezh2*^*hu5670*/*hu5670*^. Scale bar is 200 μm. (**c**) Immunostaining for Ezh2 in wildtype and *MZezh2*^*hu5670*/*hu5670*^ embryos at 1 dpf. Ezh2 shows representative nuclear localization in the forebrain of wildtype embryos and is lost in *MZezh2*^*hu5670*/*hu5670*^ embryos. Scale bar is 10 μm. (**d**) Immunostaining for H3K27me3 in wildtype and *MZezh2*^*hu5670*/*hu5670*^ embryos at 1 dpf. H3K27me3 shows representative nuclear localization in the tail of wildtype embryos and is lost in *MZezh2*^*hu5670*/*hu5670*^ embryos. Scale bar is 10 μm. (**e**) *In situ* hybridization for *hoxa9b*, *pax2*, and *shh* mRNA in *MZezh2*^*hu5670*/+^ and *MZezh2*^*hu5670*/*hu5670*^ embryos at 1 and 2 dpf. In *MZezh2*^*hu5670*/+^ embryos a clear boundary of *hoxa9b* expression is visible (arrow head) as well as expression in the pectoral fin buds (arrows). Expression is shifted to anterior in *MZezh2*^*hu5670*/*hu5670*^ embryos (arrow head). The expression pattern of *hoxa9b* in *MZezh2*^*hu5670*/+^ resembles that of wildtype embryos^54^. Scale bar is 200 μm. In *MZezh2*^*hu5670*/+^ embryos expression of *pax2* is normal and amongst others restricted to the optic stalk, mid-hindbrain boundary, and the spinal cord neurons^32^. Expression in the optic stalk is spread throughout the eye in *MZezh2*^*hu5670*/*hu5670*^ embryos. Expression of *shh* is comparable to wildtype embryos in *MZezh2*^*hu5670*/+^ embryos at 1 and 2 dpf^31^. In *MZezh2*^*hu5670*/*hu5670*^ embryos, expression of *shh* is outside the regular boundaries in the head region (arrow head) at 1 dpf and is still present at 2 dpf in the notochord, in contrast to *MZezh2*^*hu5670*/+^ embryos (arrow head). Scale bar is 500 μm. The numbers indicate the number of embryos with the displayed phenotype compared to the total number of embryos analyzed.

From an ENU-mutagenized library, a pre-mature stop mutation in *ezh2* (*hu5670*) was identified (Supplementary Fig. S1)^29^. As shown in Fig. 1b, *ezh2* mRNA is maternally provided. To study the effect of a complete loss of Ezh2 function on early development, we additionally eliminated the maternally provided *ezh2* mRNA. In zebrafish, this can be achieved through germ cell transplantations (Fig. 2a)^30^. Surprisingly, we were able to generate fertile males and females carrying *ezh2* mutant germ cells. *In situ* hybridization for *ezh2* showed that maternal contribution as well as zygotic expression of *ezh2* was indeed lost in maternal zygotic *ezh2* (*MZezh2*) mutants (Fig. 2b). In heterozygous siblings maternal transcripts are also absent, while zygotic expression of *ezh2* mRNA is present at around 50% epiboly (Fig. 2b). We subsequently investigated the presence of Ezh2 and H3K27me3 by immunohistochemistry. At 1 dpf Ezh2 and H3K27me3 are clearly detectable in wildtype embryos, while both are undetectable in *MZezh2* mutants (Fig. 2c,d). Together these data indicate that *ezh2*(*hu5670*) is a strong loss of function allele.

Since *hox, pax,* and *shh* genes are well-known targets of PcG proteins, we investigated whether these transcripts were differentially expressed in *MZezh2* mutants. Indeed, the clear boundary of *hoxa9b* expression is shifted anteriorly in *MZezh2* mutant embryos at 1 dpf (Fig. 2e, Supplementary Fig. S2). In addition, expression of *pax2* was no longer restricted to the optic stalk, but was present in the entire eye. Expression of *shh* was also observed outside the regular boundaries of expression at 1 dpf. At 2 dpf *shh* expression was prolonged and still visible in the notochord in *MZezh2* mutants, while this is not observed in heterozygous siblings. Interestingly, zebrafish embryos that lack maternal *ezh2*, but do express zygotic *ezh2*, display normal spatiotemporal expression patterns for *hoxa9b, pax2,* and *shh* (Fig. 2e)^28^,^31^,^32^, indicating that zygotic *ezh2* expression can rescue the loss of maternal *ezh2* during embryonic patterning. Consistent with this, animals lacking only maternally provided *ezh2* are viable and fertile (data not shown).

**Figure 3:**
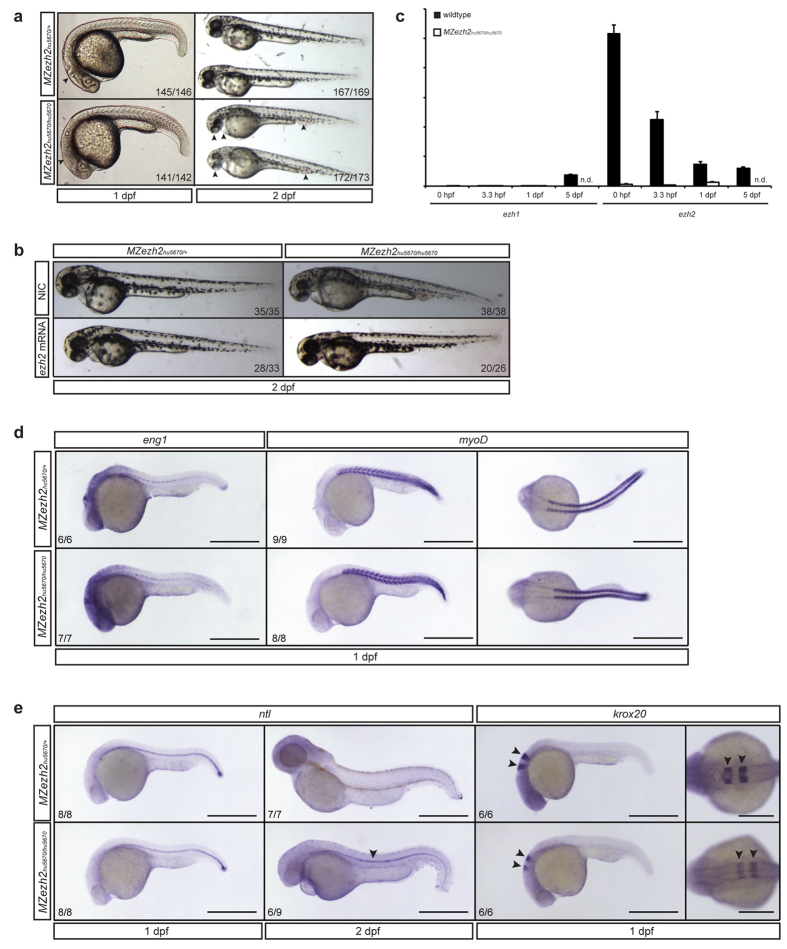
*Maternal zygotic ezh2* mutants form a normal body plan and display a pleiotropic phenotype at 2 dpf. (**a**) *MZezh2*^*hu5670*/*hu5670*^ appear relatively normal at 1 dpf, although a clear mid-hindbrain boundary appears to be absent (arrow head). They display a pleiotropic phenotype at 2 dpf, having small eyes, a stringy heart, and blood accumulation (arrow heads). *MZezh2*^*hu5670*/+^ show normal development. (**b**) The pleiotropic phenotypes of *MZezh2*^*hu5670*/*hu5670*^ can be rescued by injection of full-length *ezh2* mRNA (300 pg). The numbers indicate the number of embryos with the displayed phenotype compared to the total number of embryos injected in two experiments. (**c)** Expression analysis of *ezh1* and *ezh2* in wildtype and *MZezh2*^*hu5670*/*hu5670*^ embryos at 0 hpf, 3.3 hpf, and 1 dpf. Expression of *ezh1* is not detectable in *MZezh2*^*hu5670*/*hu5670*^ embryos and wildtype embryos at 0 hpf, 3.3 hpf, and 1 dpf. *ezh1* is expressed in wildtype control embryos at 5 dpf. *ezh2* is expressed in wildtype embryos at 0 hpf, 3.3 hpf, 1 dpf, and 5 dpf, showing a decrease in expression over time. *ezh2* expression cannot be detected in *MZezh2*^*hu5670*/*hu5670*^ embryos. Relative expression was calculated based on expression of housekeeping genes *β-actin* and *ef1α*. Error bars represent standard deviation. n.d. is not done. (**d**) *In situ* hybridization for *eng1* (muscle pioneer marker) and *myoD* (somite marker) at 1 dpf in *MZezh2*^*hu5670*/*hu5670*^ embryos and *MZezh2*^*hu5670*/+^. Both *eng1* and *myoD* are normally expressed in *MZezh2*^*hu5670*/*hu5670*^ and *MZezh2*^*hu5670*/+^. Scale bar is 500 μm. (**e**) *In situ* hybridization for *ntl* at 1 dpf shows no difference in spatiotemporal expression between *MZezh2*^*hu5670*/*hu5670*^ embryos and the heterozygous siblings. At 2 dpf *in situ* hybridization for *ntl* showed expression in the notochord of *MZezh2*^*hu5670*/*hu5670*^ embryos, whereas this is not visible in *MZezh2*^*hu5670*/+^ (arrow head). *In situ* hybridization for *krox20* at 1 dpf showed normal expression in *MZezh2*^*hu5670*/+^, but reduced expression in rhombomeres 3 and 5 in *MZezh2*^*hu5670*/*hu5670*^ embryos (arrow heads). Scale bar is 500 μm for lateral views and 250 μm for dorsal view of *krox20* expression. The numbers indicate the number of embryos with the displayed phenotype compared to the total number of embryos analyzed.

*MZezh2* mutant embryos complete gastrulation and appear to have a normal gross body plan at 1 dpf (Fig. 3a). However, these embryos seem to lack a clear mid-hindbrain boundary, even though *pax2* expression is present at this region (Fig 2e). At 2 dpf *MZezh2* mutant embryos display a pleiotropic phenotype, including small eyes, accumulation of blood near the yolk extension, a stringy heart, heart edema, and absence of pectoral fins (Fig. 3a). To determine whether these phenotypes are caused by the loss of *ezh2*, *ezh2* mRNA was injected into one-cell-stage *MZezh2* mutants and heterozygous siblings. At 2 dpf, *ezh2* mRNA-injected *MZezh2* mutants were phenotypically indistinguishable from the heterozygous siblings, evidenced by normally sized eyes and normal circulation of the blood (Fig. 3b). This indicates that the observed pleiotropic phenotype is a specific result from the loss of *ezh2*.

Since Ezh1 could potentially take over part of the function of Ezh2, we addressed the expression of *ezh1*. Until 1 dpf we could not detect *ezh1* by qPCR in *MZezh2* mutants and wildtype control embryos (Fig. 3c), indicating that during the first 24 hours of development, *MZezh2* mutants most likely lack all H3K27 trimethylation activity.

To gain information about developmental processes in the *MZezh2* mutants, we performed spatiotemporal expression analyses for *eng1* (muscle pioneer marker), *myoD* (myogenic differentiation marker), *ntl* (mesodermal marker), and *krox20* (neural marker). *eng1*, *myoD*, and *ntl* all show expression patterns comparable to expression in heterozygous sibling and wildtype embryos at 1 dpf (Fig. 3d,e, Supplementary Fig. S2)^33^, indicating that muscle tissue is formed and can differentiate in *MZezh2* mutants. However, like for *shh* we observed sustained expression of *ntl* in the notochord of *MZezh2* mutant embryos at 2 dpf (Fig. 3e), which was not observed in heterozygous siblings and wildtype embryos (Fig. 3e, Supplementary Fig. S2). In addition, expression of *krox20* appeared to be less prominent in both rhombomere 3 and 5 in *MZezh2* mutant embryos compared to heterozygous siblings and wildtype embryos (Fig. 3e, Supplementary Fig. S2).

These data surprisingly demonstrate that various cellular lineages are properly specified in absence of Ezh2 activity. Interestingly, soon after the body plan has been established, Ezh2 is required for further differentiation of cells in different tissues.

### Ezh2 affects the load of maternal mRNA in zygotes

The above results clearly demonstrate that *ezh2* is maternally provided and indicate that it has profound effects on zebrafish development, even though *MZezh2* mutant embryos survive gastrulation and are able to develop a grossly normal body plan. Given that ZGA occurs around MBT, maternally provided *ezh2* may affect gene expression during the first hours of development or in the oocyte, while resulting in detectable phenotypes much later.

**Figure 4:**
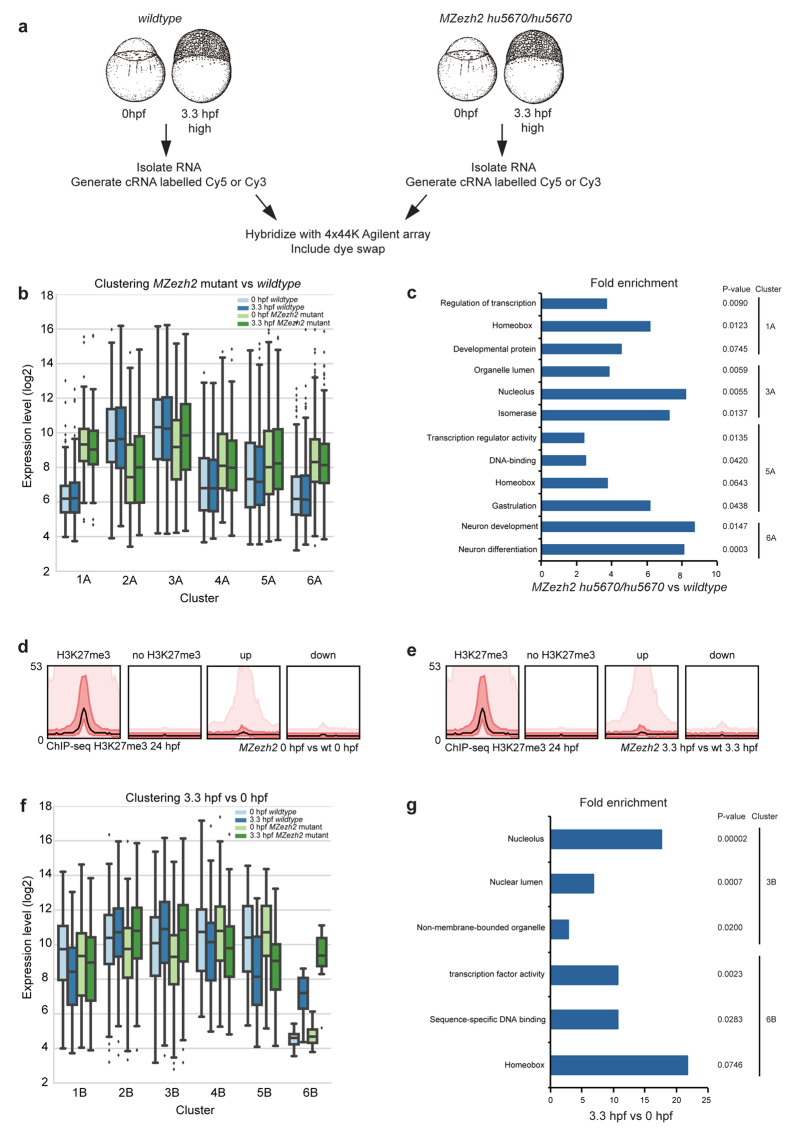
Gene expression analysis of maternal zygotic *ezh2* mutants. (**a**) Schematic overview of samples that were used for microarrays and the subsequent workflow. (**b**) Boxplots of gene expression levels (log2) for genes in cluster 1A–6A, comparing genes that are significantly differently expressed between wildtype versus *MZezh2*^*hu5670*/*hu5670*^ embryos at 0 hpf and 3.3 hpf. In comparison, expression level (log2) of housekeeping genes *actb*, *eef1a1*, and *tuba* is between 7.3 and 9.3. The mean expression (log2) of the array is between 9.5 and 10.1. (**c**) DAVID analysis on genes differently expressed between *MZezh2*^*hu5670*/*hu5670*^ and wildtype embryos at 0 hpf and 3.3 hpf. The fold enrichment of different terms is shown for the different clusters shown in Fig. 4b (Bonferroni corrected p-value  < 0.1). (**d**) Bandplots of H3K27me3 ChIP-sequencing showing presence of H3K27me3 at genes that are significantly (>2-fold, p < 0.01) up- or downregulated in *MZezh2*^*hu5670*/*hu5670*^ versus wildtype embryos at 0 hpf. The graphs show transcription start site ± 20 kb. The left panel shows the intensity distribution of the H3K27me3 peaks in wildtype embryos at 24 hpf. The mean of the median is depicted as a black line, 50% is red, and 90% is pink. (**e**) Bandplots like in Fig. 4d for genes that are significantly up- or downregulated in *MZezh2* mutant versus wildtype embryos at 3.3 hpf. (**f**) Boxplots of gene expression levels (log2) for genes in cluster 1B–6B (Supplementary Fig. S4), comparing genes that are significantly differently expressed between 0 hpf versus 3.3 hpf in wildtype and *MZezh2*^*hu5670*/*hu5670*^ embryos. (**g**) DAVID analysis on genes differently expressed between 3.3 hpf and 0 hpf in *MZezh2*^*hu5670*/*hu5670*^ and wildtype embryos. The fold enrichment of different terms is shown for the different clusters shown in Fig. 4f (Bonferroni corrected p-value < 0.1).

To address this we analyzed gene expression in wildtype and *MZezh2* mutant embryos at 0 hpf (zygote) and 3.3 hpf (MBT) using an Agilent 4 × 44K array (Fig. 4a). We identified pronounced differences in gene expression between wildtype and *MZezh2* mutant zygotes already at 0 and 3.3 hpf (Fig. 4b). Overall, 654 genes are >2-fold higher expressed in *MZezh2* mutants versus wildtype and 627 genes are >2-fold lower expressed (Fig. 4b, p < 0.01) at 0 hpf. In addition, 625 genes are upregulated and 206 downregulated in *MZezh2* mutants versus wildtype at 3.3 hpf (>2-fold, p < 0.01, Fig. 4b). The differentially expressed genes were divided into 6 clusters using pam with the Euclidean distance metric (Fig. 4b). Clusters 1A and 4A–6A contain genes that are upregulated in *MZezh2* mutant embryos compared to wildtype embryos at 0 hpf and 3.3 hpf. (Fig. 4b, Supplementary Fig. S3). To identify enriched biological themes (particularly GO terms) among these genes, we performed DAVID analysis. We identified significant enriched gene functions in cluster 1A, 3A, 5A, and 6A. This analysis indicated that genes upregulated in *MZezh2* mutants compared to wildtype (cluster 1A, 5A, and 6A) are overrepresented for developmental gene functions (Fig. 4c), including previously described Ezh2 targets like *hox*, *pax*, and *tbx* (Supplementary Table S1). Genes that are downregulated in *MZezh2* mutants compared to wildtype embryos (cluster 3A) are enriched for biological themes including organelle lumen, nucleolus, and isomerase.

Since a clear myocardial phenotype in the *MZezh2* mutants was observed, the expression of myocardial genes was analyzed in more detail. At both 0 hpf and 3.3 hpf we detected a tendency for myocardial markers to be higher expressed in *MZezh2* mutant embryos compared to wildtype embryos (Supplementary Fig. S4).

To determine whether the differentially regulated genes in *MZezh2* mutants are indeed enriched for Ezh2 targets, we compared the genes that are up- and downregulated between *MZezh2* mutant and wildtype embryos at 0 hpf and 3.3 hpf with previously published ChIP-sequencing data for H3K27me3 at 24 hpf^34^. We observed that the genes that are upregulated in *MZezh2* mutants at 0 hpf and 3.3 hpf are enriched for H3K27me3 peaks under normal conditions (Fig. 4d,e), while such enrichment is not seen for downregulated genes. This suggests that upregulation is due to direct effect of loss of Ezh2 activity and that downregulation most likely stems from indirect effects.

We hypothesize that *ezh2* is involved in placing epigenetic signatures during oogenesis that in turn are translated into the establishment of a proper maternal mRNA load of the zygote. This includes mRNAs that do not have a clear role within the oocyte itself, but only function after fertilization, and emphasizes the importance of *ezh2* in transmitting epigenetic information through the transmission of maternal mRNAs.

### Ezh2 affects gene expression during early embryonic development

In order to determine how gene expression is regulated over time, we continued to assess how gene expression changes between 0 hpf and 3.3 hpf, and how this is affected in *MZezh2* mutant embryos (Fig. 4f, Supplementary Fig. S3). Overall, the changes in gene expression seem to follow the same pattern from 0 hpf to 3.3 hpf in both wildtype and *MZezh2* mutant embryos. However, a proportion of transcripts in cluster 1B are downregulated over time in wildtype embryos but not in *MZezh2* mutant embryos. In addition, transcripts in cluster 2B show little change in expression in wildtype embryos between 0 hpf and 3.3 hpf, while they become more abundant at 3.3 hpf compared to 0 hpf in *MZezh2* mutants (Fig. 4f, Supplementary Fig. S3). This suggests that expression of genes in clusters 1B and 2B is normally controlled in a temporal manner by Ezh2. Furthermore, genes in cluster 3B and 6B are upregulated in both wildtype and *MZezh2* mutants from 0 hpf to 3.3 hpf, but the difference in expression is larger in *MZezh2* mutants.

We performed DAVID analysis on these genes to identify enriched biological themes on genes that are differently expressed between 0 hpf and 3.3 hpf in wildtype and *MZezh2* mutants (Fig. 4g). We only identified significantly enriched gene functions in cluster 3B and 6B (Fig. 4g). As indicated above, both clusters are more upregulated in *MZezh2* mutants compared to wildtype embryos at 3.3 hpf and are therefore potential targets of Ezh2. Genes in these clusters are involved in nucleolus, nuclear lumen, non-membrane bounded organelle, transcription factor activity, sequence-specific binding, and also include homeobox genes (Supplementary Table S2).

Together, these analyses reveal that Ezh2 not only dictates the maternal load of mRNAs, but also affects the transcription of genes during early embryonic development.

### Expression of myocardial markers in MZezh2 mutants

**Figure 5:**
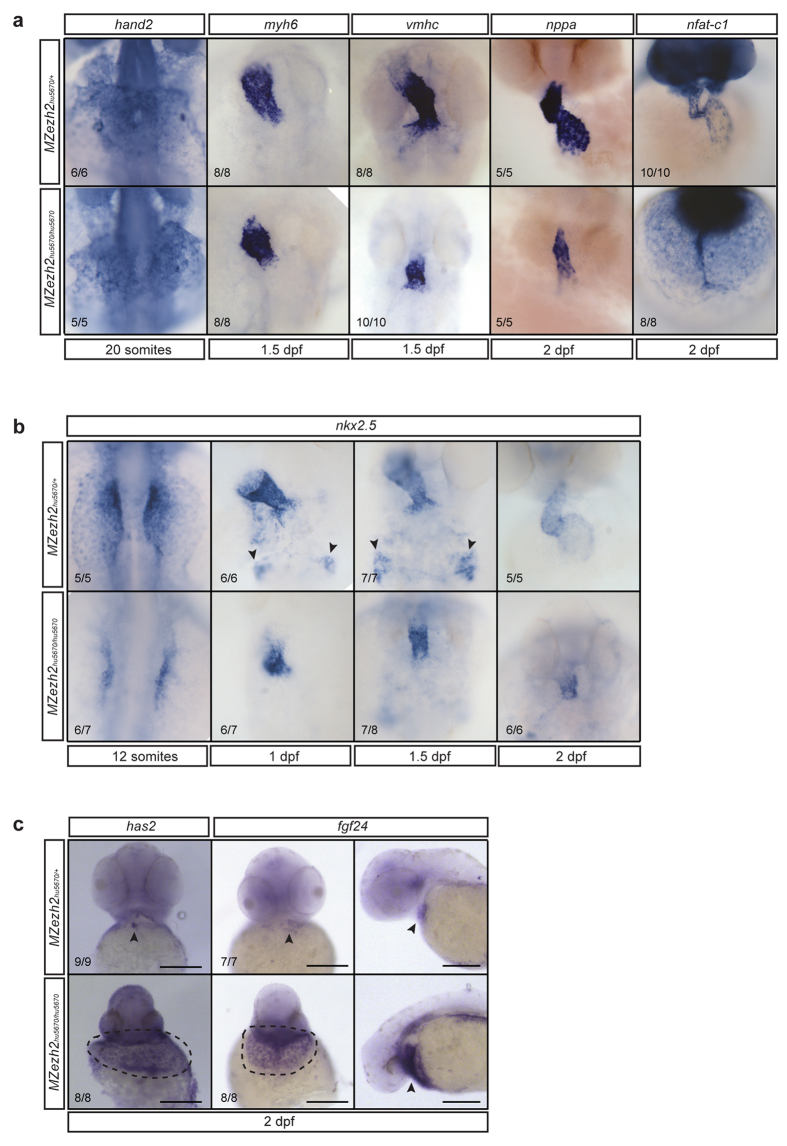
Myocardial development is affected in *MZezh2* embryos. (**a**) *In situ* hybridization for different heart markers in *MZezh2*^*hu5670*/+^ and *MZezh2*^*hu5670*/*hu5670*^ at various time points of development. *hand2* is an early myocardial marker. *myh6* is a marker for atrial cells. *vmhc* is a marker for ventricular cells. *nppa* is a late myocardial marker. *nfat-c1* is an endocardial marker. All these markers are expressed in *MZezh2*^*hu5670*/*hu5670*^, although *vmhc*, *nppa,* and *nfat-c1* expression show a smaller number of positive cells. (**b**) *In situ* hybridization for *nkx2.5* at different time points after fertilization in *MZezh2*^*hu5670*/+^ and *MZezh2*^*hu5670*/*hu5670*^ embryos. Arrow heads point to cells of the pharyngeal arch artery progenitors. This is absent in *MZezh2*^*hu5670*/*hu5670*^. (**c**) *In situ* hybridization for *has2* and *fgf24* at 2 dpf in *MZezh2*^*hu5670*/*hu5670*^ and their heterozygous siblings. In *MZezh2*^*hu5670*/+^ expression is restricted to the heart (arrow heads), whereas in the *MZezh2*^*hu5670*/*hu5670*^ embryos expression is visible in the area surrounding the heart tube (encircled by dashed line). For *fgf24* this is also shown from a lateral view (arrow heads). Scale bar is 200 μm. The numbers indicate the number of embryos with the displayed phenotype compared to the total number of embryos analyzed.

Next, we aimed to better understand the origin of the pleiotropic defects observed in *MZezh2* embryos. Since *MZezh2* mutants develop a ‘stringy-heart’, which was one of the most pronounced phenotypes, cardiac development was studied in more detail. To gain knowledge about the specification and differentiation of various cardiac lineages, *in situ* hybridization for different cardiac markers was performed (Fig. 5a–c). Morphologically, the heart fails to undergo cardiac looping resulting in a straight heart tube at 2 dpf in *MZezh2* mutant embryos (Fig. 5a, Supplementary Fig. S5). Expression analysis for *vmhc* revealed a smaller ventricle in *MZezh2* mutants compared to heterozygous siblings at 1.5 dpf (Fig. 5b). Next to *vmhc*, we analyzed expression of *hand2* (early marker), *myh6* (atrial marker), *nppa* (late marker), and *nfat-c1* (endocardial marker) and showed that these markers are all expressed in the *MZezh2* mutant (Fig 5a).

To continue the analyses, we studied expression of *nkx2.5*, a homeodomain transcription factor and an early myocardial marker. This marker is readily expressed starting at the 12-somite stage in wildtype embryos, but the area of *nkx2.5* expression seems to be smaller in the *MZezh2* mutant at this stage (Fig. 5b). Also later during development we observed a smaller region of *nkx2.5* expressing cells in *MZezh2* mutants (Fig. 5b). Interestingly, the posterior group of *nkx2.5* positive cells, the pharyngeal arch artery progenitors^35^,^36^, is absent in *MZezh2* mutants at 1 and 1.5 dpf (Fig. 5a). We conclude from these experiments that, while cardiac cell numbers may be affected in *MZezh2* mutants, general differentiation markers for different compartments of the heart tube are grossly expressed normally.

Even though we observed that the above-mentioned myocardial markers are expressed grossly normally, this is not valid for all markers. To start with, the developmental and atrioventricular canal marker *has2* is normally and specifically expressed in the heterozygous siblings at 2 dpf. In contrast, in *MZezh2* mutant embryos we observe ectopic expression of *has2* (Fig. 5c). The same observation was made for *fgf24* (Fig. 5c). This gene is downstream of *tbx5*, a transcription factor essential for heart and limb formation^37^,^38^ and *fgf24* was upregulated in our expression study (Supplementary Table S1). Whereas *fgf24* expression is spatially restricted in heterozygous siblings, a broad ring of expression around the heart tube was observed in *MZezh2* mutant embryos (Fig. 5c). Similar results were obtained when performing expression analysis for myocardial markers *myl7* and *mef2cb* at 2 dpf (Supplementary Fig. S5). Overall, these results suggest that myocardial cells are specified, but seem to get dispersed over an area around the regular heart tube over time.

### MZezh2 mutants display a loss of myocardial tissue integrity

**Figure 6:**
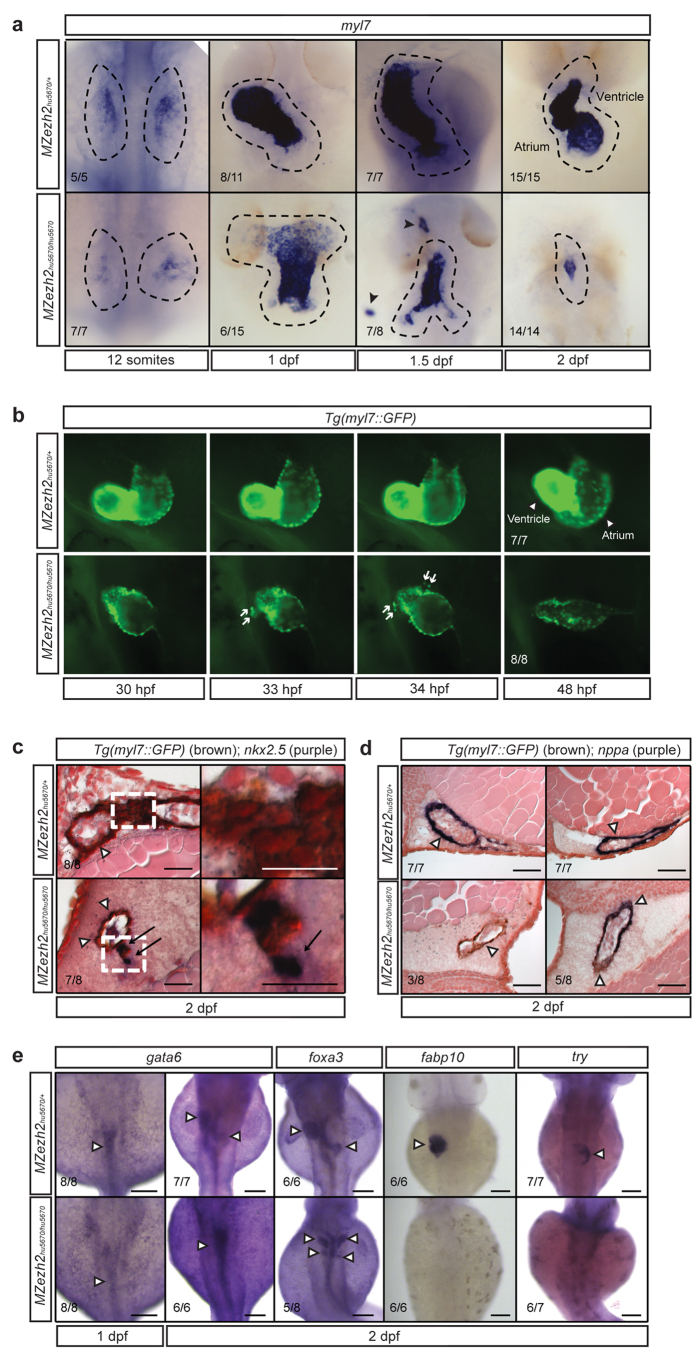
*MZezh2* mutant embryos display impaired myocardial and gastrointestinal tissue maintenance. (**a**) *In situ* hybridization for *myl7* at different time points in *MZezh2*^*hu5670*/+^ and *MZezh2*^*hu5670*/*hu5670*^. At 1 dpf the heart of *MZezh2*^*hu5670*/*hu5670*^ is straight. In *MZezh2*^*hu5670*/*hu5670*^ embryos *myl7* expressing cells are found outside the heart at 1.5 dpf (arrow heads). At 2 dpf the number of *myl7* expressing cells is decreased in *MZezh2*^*hu5670*/*hu5670*^ compared to *MZezh2*^*hu5670*/+^. (**b**) Stills of time lapse (Supplementary Movie S1,2) imaging of *Tg(myl7::GFP) MZezh2*^*hu5670*/+^ and *MZezh2*^*hu5670*/*hu5670*^ embryos from 1 to 2 dpf. In *MZezh2*^*hu5670*/*hu5670*^ embryos, GFP-positive cells are moving away from the heart (arrows). Arrow heads point at ventricle and atrium. (**c**) Immunostaining for GFP to visualize *Tg(myl7::GFP)* (brown precipitation, arrow heads) combined with *in situ* hybridization for *nkx2.5* (purple staining, arrows) at 2 dpf. In *MZezh2*^*hu5670*/+^ no *nkx2.5* expressing cells are present. In *MZezh2*^*hu5670*/*hu5670*^ cells that are detached from the heart express *nkx2.5*. The right panel shows a zoom-in of the left panel (white square). Scale bar is 50 μm. (**d**) Immunostaining for GFP *Tg*(*myl7::GFP*) combined with *in situ* hybridization for *nppa* at 2 dpf in *MZezh2*^*hu5670*/+^ and *MZezh2*^*hu5670*/*hu5670*^ embryos. Two embryos per genotype are shown. In *MZezh2*^*hu5670*/*hu5670*^ embryos *nppa* expression is absent in one and not ubiquitous in the other embryo (arrow heads). In *MZezh2*^*hu5670*/+^ *nppa* is expressed in atrium and ventricle (arrow heads). Scale bar is 50 μm. (**e**) *In situ* hybridization for different gastrointestinal tract markers at 1 and 2 dpf in *MZezh2*^*hu5670*/*hu5670*^ and *MZezh2*^*hu5670*/+^. Expression of *gata6* is present in both *MZezh2*^*hu5670*/*hu5670*^ and *MZezh2*^*hu5670*/+^ at 1 and 2 dpf. At 2 dpf the intestinal tube appears straight in the *MZezh2*^*hu5670*/*hu5670*^, whereas structures like the liver and pancreas can be seen in *MZezh2*^*hu5670*/+^ (arrow heads). *MZezh2*^*hu5670*/*hu5670*^ are able to form a gastrointestinal tract, observed by *in situ* hybridization for *foxa3* at 2 dpf, although the organs are bilaterally formed (arrow heads). In *MZezh2*^*hu5670*/*hu5670*^ no expression of terminal differentiation markers for liver, *fabp10,* and exocrine pancreas, *try,* was observed. Scale bar is 100 μm. Numbers indicate the number of embryos with the displayed phenotype compared to the total number of embryos analyzed.

To further investigate the morphogenetic changes that establish the heart tube over time we performed a time course of *in situ* hybridization analysis for *myl7* (Fig. 6a). At the 12-somite stage, myocardial precursors are present in *MZezh2* mutants, even though their location and number appears to be slightly affected as shown by *nkx2.5* and *hand2* expression analysis (Fig. 5a,b). At 1 dpf the heart of heterozygous siblings starts to jog to the left, like in wildtype embryos, while the heart of *MZezh2* mutants frequently remains straight (Supplementary Fig. S5). Despite the lack of jogging, a heart tube is still formed (Fig. 6a,b). At 1.5 dpf the heart of heterozygous siblings starts to undergo cardiac looping (Fig. 6a). This bending of the heart tube did not occur in *MZezh2* mutant embryos (Fig. 6a). Remarkably, at this stage *myl7* expressing cells are visible outside the heart tube and the heart appears to be smaller in size in *MZezh2* mutants (Fig. 6a). At 2 dpf only a small tube of *myl7* expressing cells remains in *MZezh2* mutants.

We next determined whether the extra-cardiac *myl7* positive cells we observed around 1.5 dpf represent cells that are derived from the original heart or they are non-heart-related cells that aberrantly start to express *myl7*. We performed time-lapse imaging on 1 to 2 dpf *MZezh2* mutants and heterozygous siblings carrying a *Tg*(*myl7::GFP*) transgene (Fig. 6b, Supplementary Movie S1,2). We observed that at around 33–34 hpf, GFP positive cells detach and move away from the heart tube. These detaching cells appear to be derived from both the ventricle and the atrium (Fig. 6b). These results indicate that the extra-cardial cells are lost from the originally formed heart. They also suggest that a loss of cardiac integrity underlies the reduction of the size of the heart tube, and that Ezh2 is required to regulate genes that maintain structural integrity of the cardiac tube.

A loss of cell adhesion may cause the loss of cells from the heart, as it is known that in *fn* morphants cardiac progenitors fail to form the cardiac disc, which results in two heart fields^39^. In addition, in mice it was shown that Ezh2 represses regulators of extracellular matrix remodeling in endothelial cells^40^. To address this, expression of dm-GRASP, a cell-surface adhesion molecule of the immunoglobulin superfamily^41^, was assessed. Immunostaining for dm-GRASP showed expression of this marker in the hearts of *MZezh2* mutants, indicating that cell adhesion is not affected in *MZezh2* mutants (Supplementary Fig. S5). In addition, we did not observe a difference in apoptosis between *MZezh2* mutant embryos compared to heterozygous siblings (Supplementary Fig. S5).

Finally, to gain insight into the identity and differentiation status of the cells that detach from the heart, we combined *in situ* hybridization for *nkx2.5* with immunostaining for GFP in a *Tg*(*myl7::GFP*) background on 1, 1.5, and 2 day old embryos. At 1 dpf there are no major differences between heterozygous siblings and *MZezh2* mutant embryos (Supplementary Fig. S5). Remarkably, at 1.5 dpf the expression of *nkx2.5* is partially lacking in *MZezh2* mutants, whereas it is expressed in heterozygous siblings (Supplementary Fig. S5). The cells of *MZezh2* mutants that lack expression of *nkx2.5* do express GFP. At 2 dpf, the cells of the heart tubes of both *MZezh2* mutants as well as heterozygous siblings are GFP (Myl7) positive but *nkx2.5* negative. However, the cells that are detached from the heart tube in *MZezh2* mutants are both GFP (Myl7) and *nkx2.5* positive (Fig. 6c). Even though GFP has a half-life of 26 hours, meaning that GFP-positive cells do not necessarily express *GFP* at the RNA level, this result strongly suggests that in total absence of Ezh2, myocardial cells fail to silence *nkx2.5*. In addition, we also combined immunostaining for GFP with *in situ* hybridization for *nppa,* a late myocardial differentiation marker. Next to observing a smaller heart tube at 2 dpf in *MZezh2* mutants (Fig 5a), we observed a partial loss of *nppa* expression in *MZezh2* mutants, whereas it was expressed throughout the heart in heterozygous siblings (Fig. 6d). This suggests a defect in terminal differentiation of *MZezh2* mutant myocardial cells, possibly related to the observed problems in properly repressing *nkx2.5*. We conclude that myocardial cells in *MZezh2* mutants likely have problems to maintain cardiac differentiation and that this may lead to the structural instability of the heart.

### Loss of ezh2 affects terminal differentiation of the liver and pancreas

To address whether this loss of tissue integrity and defects in terminal differentiation is specific for the heart we also addressed cell differentiation in other tissues. For this we chose the gastrointestinal tract and the associated organs. Expression analysis for *gata6*, an early endoderm marker, showed normal expression at 1 dpf in *MZezh2* mutants. The gut of *MZezh2* mutants is straight at 2 dpf based on *gata6* expression, whereas it has looped in heterozygous siblings (Fig. 6e). Interestingly, expression of *foxa3*, a definite marker of endoderm, showed incorrect looping or a bilateral gastrointestinal tract in *MZezh2* mutants (Fig. 6e). Finally, *in situ* hybridization for the terminal differentiation markers for liver and the exocrine pancreas, *fabp10* and *try* respectively, revealed a loss of expression suggesting that formation of these organs is delayed or abrogated in *MZezh2* mutant embryos (Fig. 6e). These results indicate that the gastrointestinal tract, including the liver and pancreas, is formed initially in *MZezh2* mutant embryos, but fails to terminally differentiate. Thus, problems in terminal differentiation in *MZezh2* mutants are not heart-specific, but different organs derived from different germ layers are affected.

## Discussion

The function of Ezh2 during development has been intensely studied using different model systems, including mouse and *Drosophila*. Despite these studies, many open questions remain regarding the developmental roles of Ezh2. Our study sheds new light on the requirement of Ezh2 during early vertebrate development. Most importantly, our results indicate that the basic vertebrate body plan can be established without Ezh2, but that Ezh2 is essential for the maintenance of a wide range of tissues, possibly by playing a role in terminal differentiation. In the following sections we will discuss possible scenarios regarding the roles of Ezh2 during vertebrate development.

### Function of Ezh2 in germ cells

Our results demonstrate a clear function for *ezh2* during embryonic development. Strikingly, even though the maternal-to-zygotic transition occurs around 3–4 hours after fertilization, the first phenotypic differences between wildtype embryos and embryos lacking both maternal and zygotic *ezh2* are not evident until hours after gastrulation. This may hint to a mechanism in which maternally expressed *ezh2* acts by pre-labeling genes with specific chromatin marks such that they can be properly regulated later during development. It is possible that without this pre-labeling, genes cannot be properly shut down after being activated, like we show for a number of myocardial markers and *shh* and *ntl*. Even though we have not timed when this activity would be required, our data suggest that this pre-labeling may in fact already occur during oogenesis. This is supported by observations in *Ring1/Rnf2* mutant mice that show that Polycomb group proteins act in the female germline to establish developmental competence^20^. Also in *C. elegans* transgenerational inheritance of H3K27me3 has been demonstrated^42^. In addition, work in *Drosophila* showed that PRC2 plays a role in determining germ cell fate^43^,^44^. We note that this maternal activity is not absolutely essential for viability, since embryos lacking only maternal *ezh2*, while expressing zygotic *ezh2,* can develop into fertile adults. Apparently, the embryo is able to handle a wide range of gene expression levels during early development.

The *ezh2* germline mutants are fertile and able to form *MZezh2* mutant embryos. The germ cells of *ezh2* germline mutants are originally derived from an incross between heterozygous parents. This implies that these germ cells, lacking zygotic expression of *ezh2,* obtained correct epigenetic labeling from the parents and this may be the reason they can function normally. Whether the germ cells of *MZezh2* mutant zebrafish embryos are functional needs to be tested by serial transplantation assays. Previous studies in mouse and human have shown that during germline development H3K27me3 is almost exclusively present at genes important for somatic development^45^,^46^, and hence ectopic expression of these genes in *MZezh2* mutant germ cells may lead to sterility. In concordance, *C. elegans* mutants for PRC2 homologs display a maternal effect sterile phenotype^47^,^48^,^49^.

### Ezh2 does not affect early zebrafish development

*MZezh2* mutant embryos lack Ezh2 and H3K27me3 and show major differences in gene expression even before the zygotic genome is activated. Still, these embryos are able to form a normal body plan and only die at a time point when tissue specification has taken place, indicating zygotic genome activation is not strongly affected. This is in contrast with Polycomb group mutants in other vertebrates, where loss of Polycomb group gene expression results in early lethality, mostly before gastrulation^5^,^6^,^19^,^20^,^50^,^51^,^52^,^53^. The reason for this ‘delayed’ lethality in zebrafish is not completely clear. One could argue that Ezh1 is able to compensate for the loss of Ezh2, since it was reported that Ezh1 can also trimethylate H3K27^16^. However, we think this is highly unlikely, since we show that *ezh1* is not maternally loaded into the zebrafish embryo, and based on our array and qPCR data, is not expressed in *MZezh2* mutant embryos until at least 1 dpf.

A potential explanation for the lack of an early developmental phenotype of *MZezh2* mutants in zebrafish is that unlike mice, zebrafish embryos do not form extra-embryonic tissue, which is essential for normal murine development. Another explanation may be found in differences in developmental timing between mice and zebrafish. In mice, maternal contribution lasts only until the 2-cell stage, while in zebrafish embryos this lasts until at least 1,000-cell stage^23^. Nevertheless, the fact that zebrafish embryos can gastrulate properly in the absence of Ezh2 indicates that this crucial developmental event does not critically depend on Polycomb gene activity. This makes the zebrafish a very interesting and unique model system to study Ezh2, and Polycomb function in general, during tissue specification and maintenance.

### Ezh2 function in tissue maintenance

Most of the defects we observed in *MZezh2* mutants relate to tissue maintenance. For example, the heart and the gastrointestinal tract can be specified but fail to be properly maintained. The observed loss of tissue maintenance does not seem to be caused by apoptosis. Alternatively, the failure of tissues to terminally differentiate might be caused by an arrest in proliferation, potentially by deregulation of genes involved in cell cycle control. Terminal differentiation defects were also observed in *rnf2* mutant zebrafish during pectoral fin and cranial facial development^28^,^54^. Although *rnf2* was only zygotically absent in these mutants and Rnf2 is part of PRC1 instead of PRC2, this indicates a common mechanism of involvement of Polycomb group genes in terminal differentiation. By more detailed studies of the developing heart tube we show that myocardial integrity cannot be maintained in the absence of Ezh2, while cell adhesion is not affected. In addition to the well-known function of Ezh2 as a suppressor of gene expression, it can also directly methylate non-histone targets. One example of this is the cardiac transcription factor GATA4, where methylation of GATA4 by PRC2 results in inhibition of GATA4 transcriptional activity in mice^55^. This function of PRC2 potentially plays a role in the observed myocardial phenotype of *MZezh2* zebrafish mutants.

Studies in mice, where conditional knockouts for *Ezh2* were generated using different heart specific promoters, showed that loss of *Ezh2* at an early time point results in cardiac defects, whereas loss of *Ezh2* after the heart is fully formed does not show a severe phenotype^9^,^10^. Possibly, there is a sensitive period during which Ezh2 represses its targets in progenitor cells to safeguard normal myocardial development, followed by terminal differentiation of myocardial cells, after which Ezh2 becomes dispensable for maintenance of silencing, because other chromatin features may stably lock gene expression status^4^,^6^,^56^,^57^.

Another mechanism through which Ezh2 may affect tissue maintenance is that Ezh2 may have a critical role within tissue-specific stem cells, such that upon loss of Ezh2 the tissue cannot be properly supported by the addition of newly differentiating cells^8^,^9^,^10^,^11^,^12^. Discrimination between these mutually non-exclusive scenarios will require the identification and study of relevant stem cell pools of the affected tissues, and tracing experiments in order to follow gene expression within single cells.

Taken together, our work implies that Ezh2 is essential for tissue maintenance and to set up proper maternal mRNA contribution, and presents a novel and powerful tool to study how Polycomb group proteins act during early vertebrate development and tissue maintenance.

## Methods

### Zebrafish genetics and strains

Zebrafish (*Danio rerio*), were housed according to standard conditions^58^ and staged according to Kimmel *et al*^59^. The *ezh2* nonsense mutant (*hu5670*, R592STOP) was derived from ENU mutagenized libraries using target-selected mutagenesis as described^29^. Zebrafish with the *ezh2* mutant allele were outcrossed against wildtype zebrafish (TL or AB) and subsequently incrossed to obtain homozygous mutants. *Tg*(*myl7::GFP*) and *Tg*(*vas::eGFP*) zebrafish have been described before^60^,^61^. All experiments were carried out in accordance with animal welfare laws, guidelines, and policies and were approved by the Utrecht University and the Radboud University Animal Experiments Committee.

### Genotyping

DNA was purified from caudal fin tissue taken from anesthetized zebrafish, or from embryos. An *ezh2* fragment was amplified by nested PCR with primers indicated in Supplementary Table S3. The *ezh2* mutation (*hu5670*, CCTGGCTGTA(C > T)GAGAGTGTGA) results in the loss of an RsaI restriction site. PCR was followed by RsaI restriction to finalize genotyping (Supplementary Fig. S2).

### Germ cell transplantation

Germ cell transplantation was performed as described previously^30^. At 4 hpf cells from the margin of the embryo were transplanted into wildtype hosts that were injected with the *dead end* morpholino, resulting in death of the primordial germ cells of the host^62^. Transplanted cells were labeled with *Tg*(*vas::eGFP*) and were derived from an *ezh2*(*hu5670*) heterozygous incross. After transplantation the donors were genotyped. At 1 dpf it was assessed whether the transplantation was successful, after which these embryos were raised to adulthood, obtaining a wildtype zebrafish harboring an *ezh2* mutant germline. The adult female germline mutants were checked for being 100% mutant by crossing them to a male germline mutant or a male *ezh2* heterozygous mutant zebrafish and determine the phenotype and genotype of the progeny. For all germline mutants used in this study the resulting progeny was 100% or 50% homozygous mutant, depending on the genotype of the zebrafish it was crossed with. The germline mutant zebrafish displayed normal fertility and produced 200–600 embryos per cross. The *MZezh2* mutant embryos all displayed the same phenotype. For the experiments below we used siblings from a cross of *ezh2* germline mutant females with heterozygous *ezh2* mutant males and genotyped them afterwards. For the gene expression analysis we crossed *ezh2* germline mutant females with *ezh2* germline mutant males to obtain 100% *MZezh2* mutant progeny. Since the *MZezh2* mutant embryos display a lethal phenotype, the embryos that were used were the first generation after germline transplantation.

### Histological analysis

Zebrafish embryos were sacrificed with Tricaine and ice-cold water, fixed overnight in 4% PFA in PBS at 4 °C. After fixation the embryos were gradually transferred to 75% ethanol after which they were embedded in plastic for sectioning. Plastic sections were stained with haemotoxylin and eosin for histological analysis.

### Whole mount *in situ* hybridization

Embryos were fixed overnight at 4 °C in 4% PFA in PBS, after which they were gradually transferred to 100% methanol. Embryos older than 24 hpf were treated with proteinase K. *In situ* hybridization was performed as described previously^63^. The embryos were imaged by light microscopy or embedded in plastic for sectioning and imaging.

### Immunostainings

Immunostainings were performed as described previously^63^,^64^. Embryos were fixed in 4% PFA in PBS at 4 °C overnight. After fixation they were gradually transferred to 100% methanol. Rabbit anti-Ezh2 antibody from Cell Signalling Technologies was used (1:200). The epitope of this antibody is located upstream of the SET domain and the identified nonsense mutation in *ezh2*. Rabbit anti-H3K27me3 antibody from Millipore was used (1:750). Cy3-anti-rabbit antibody from Jackson ImmunoResearch was used as secondary antibody. Immunostainings were analyzed using a confocal fluorescent microscope (Leica, SP5). Immunostainings after *in situ* hybridization and for dm-GRASP and active Caspase-3 were performed with a rabbit anti-GFP from Gentaur (1:200), mouse anti-dm-GRASP from DSHB (1:200), and anti-Caspase-3 from BD Biosciences (1:500), respectively, followed by a peroxidase labeled polymer (Immunovision and Dako) for DAB staining. The immunostainings were analyzed using a light microscope. When embedded in paraffin, the sections were stained with neutral red.

### qPCR analysis

Total RNA was isolated from 0 hpf, 3.3 hpf, and 1 dpf wildtype and *MZezh2* mutant embryos using Trizol. cDNA was synthesized using Superscript II (Invitrogen). Standard qPCR using SYBR Green was performed using the primers shown in Supplementary Table S4. Relative expression was corrected for primer efficiency and calculated based on expression of housekeeping genes *β-actin* and *ef1α*.

### Time lapse imaging

Embryos of 1 dpf were dechorionated and mounted in glass bottom plates using 0.25% agarose in E3 embryo medium containing Tricaine. Confocal imaging was performed overnight using a LEICA AF7000 microscope. Pictures were taken with 7.5-minute intervals.

### Gene expression microarrays

Custom 4 × 44k microarrays for zebrafish from Agilent were used according to manufacturer’s protocol. 200 ng of total RNA from 1 cell stage embryos and embryos of 3.3 hpf was converted into cRNA and labeled with Cy3 or Cy5. Samples were subsequently hybridized overnight and washed. A dye swap was included as a technical replicate. The experiment was performed in duplicate using biological replicates. The arrays were processed using R/Bioconductor and limma^65^. After background correction, within-array normalization (loess) and between-array normalization (Aquantile) was performed. Differential expression was determined using eBayes method. The expression profiles were clustered using pam with the Euclidean distance metric. We used the biomaRt package^66^,^67^ to provide the Ensembl annotation with systematic name and genomic location based on the probe identifiers.

H3K27me3 ChIP-seq data for 24 hpf was obtained from NCBI GEO (GSE35050^34^) and mapped to the zebrafish genome (danRer7/Zv9) with bwa^68^. The bandplots were created using fluff^69^ for the transcription start sites of differentially expressed genes (fold change >=2) and genes present on the array with or without H3K27me3 enrichment. DAVID annotation^70^,^71^ was obtained from https://david.ncifcrf.gov/.

The data discussed in this publication have been deposited in NCBI’s Gene Expression Omnibus and are accessible through GEO Series accession number GSE64618 (https://www.ncbi.nlm.nih.gov/geo/query/acc.cgi?acc=GSE64618).

## Additional Information

**How to cite this article**: San, B. *et al.* Normal formation of a vertebrate body plan and loss of tissue maintenance in the absence of *ezh2. Sci. Rep.* **6**, 24658; doi: 10.1038/srep24658 (2016).

## Supplementary Material

Supplementary Information

Supplementary Movie 1

Supplementary Movie 2
