# Supplementary material for: Normal formation of a vertebrate body plan and loss of tissue maintenance in the absence of ezh2
Source: Sci Rep. 2016 May 5;6:24658. doi: 10.1038/srep24658 (PMC4857124; doi:10.1038/srep24658)
Supplement: Supplementary Information [file srep24658-s1.pdf]

SUPPLEMENTARY INFORMATION

# Normal formation of a vertebrate body plan and loss of tissue maintenance in the absence of *ezh2*

AUTHORS

Bilge San[1$], Naomi D. Chrispijn[2$], Nadine Wittkopp[3,4], Simon J. van Heeringen[5], Anne K. Lagendijk[3#], Marco Aben[1], Jeroen Bakkers[3,6], René F. Ketting[3,4], Leonie M. Kamminga[1,2,3*]

AFFILIATIONS

[1]Radboud University Medical Center, Radboud Institute for Molecular Life Sciences, Nijmegen, The Netherlands, [2]Radboud University, Faculty of Science, Department of Molecular Biology, Radboud Institute for Molecular Life Sciences, Nijmegen, The Netherlands, [3]Hubrecht Institute, University Medical Centre Utrecht, Utrecht, The Netherlands, [4]Institute of Molecular Biology, Mainz, Germany, [5]Radboud University, Faculty of Science, Department of Molecular Developmental Biology, Radboud Institute for Molecular Life Sciences, Nijmegen, The Netherlands, [6]Medical Physiology, University Medical Centre Utrecht, Utrecht, The Netherlands.

[#]Present address: University of Queensland, Institute for Molecular Biosciences, Australia.

*Corresponding author: l.kamminga@science.ru.nl

[$]These authors contributed equally to the work

SUPPLEMENTARY FIGURE LEGENDS

**Fig. S1.** Alignment of *ezh2* orthologs of zebrafish, human, mouse, and *Drosophila*. Red bar indicates the SET domain, blue bar indicates the WD domain. *hu5670* (C>T) is indicated in green. 5'- CCTGGCTGTA (C>T) GAGAGTGTGA -3' R->stop. Asterisk indicates positions that have a single, fully conserved residue. A colon indicates conservation between groups of strongly similar properties - scoring > 0.5 in the Gonnet pam 250 matrix. A period indicates conservation between groups of weakly similar properties - scoring =< 0.5 in the Gonnet pam 250 matrix.

**Fig. S2. a.** Immunostaining for Ezh2 at 4 cells, 4 hpf, and 12 somites in wildtype embryos. Expression of Ezh2 (brown precipitation) is visible at 12 somites. Scale bar is 200 μm. **b.** *In situ* hybridization for *pax2* 1 and 2 dpf and *shh* at 2 dpf in *MZezh2*$^{hu5670/hu5670}$ embryos and *MZezh2*$^{hu5670/+}$. Expression of *pax2* in *MZezh2*$^{hu5670/hu5670}$ embryos appears to be more posterior at 2 dpf and there is no clear mid-hindbrain boundary visible. Expression of *shh* at 2 dpf shows normal patterns in *MZezh2*$^{hu5670/+}$ embryos (dorsal view). In *MZezh2*$^{hu5670/hu5670}$ embryos, *shh* expression still present in the notochord at 2 dpf, in contrast to *MZezh2*$^{hu5670/+}$ embryos (arrow head). Scale bar is 500 μm. **c.** *In situ* hybridization for *hoxd9a*, *hoxc8a*, and *hoxc6a* in *MZezh2*$^{hu5670/+}$ and *MZezh2*$^{hu5670/hu5670}$ embryos at 1 dpf. The expression pattern of these hox genes in *MZezh2*$^{hu5670/+}$ resembles that of wildtype embryos (van der Velden et al., 2013). *MZezh2*$^{hu5670/hu5670}$ embryos show that the boundary of *hox* expression is shifted to anterior (arrow heads). Scale bar is 200 μm. **d.** *In situ* hybridization for *eng1*, *ntl*, and *krox20* at 1 dpf and for *ntl* at 2 dpf in wildtype embryos. Scale bar is 500 μm. **e.** Genotyping results from an example of an *ezh2* heterozygous incross at 2 dpf. After nested PCR a product of 620 bp is formed. Subsequent restriction with RsaI, of which one site is absent in the *ezh2* mutant, results in the distinct patterns for wildtype, heterozygous, and mutant embryos (bands of 281, 224, 159, 122, and 57 bp). The samples were run on a 3% agarose gel. The numbers at the *in*

*situ* hybridization and immunostaining indicate the number of embryos with the displayed phenotype compared to the total number of embryos analyzed.

**Fig. S3. a.** Cluster analysis of genes significantly differentially expressed in *MZezh2*$^{hu5670/5670}$ versus wildtype embryos at 0 hpf and 3.3 hpf. **b.** Gene expression levels (log2) of genes significantly differentially expressed between wildtype and *MZezh2*$^{hu5670/5670}$ embryos at 0 hpf. Genes in different clusters (see Fig. 4b) are depicted in different colors. **c.** Same as in Fig. S3b, for genes that are significantly differentially expressed between wildtype and *MZezh2*$^{hu5670/5670}$ embryos at 3.3 hpf. **d.** Cluster analysis of genes significantly differentially expressed between 0 hpf and 3.3 hpf in *MZezh2*$^{hu5670/5670}$ and wildtype embryos. **e.** Gene expression levels (log2) of genes significantly differentially expressed between 0 hpf and 3.3 hpf in wildtype and *MZezh2*$^{hu5670/5670}$ embryos. Genes in different clusters (see Fig. 4f) are depicted in different colors. **f.** Same as in Fig. S3e, for genes that are significantly differentially expressed between 0 hpf and 3.3 hpf in *MZezh2*$^{hu5670/5670}$ embryos.

**Fig. S4. a.** Gene expression levels (log2) of a number of known myocardial markers in wildtype versus *MZezh2*$^{hu5670/5670}$ embryos at 0 hpf. **b.** Gene expression levels (log2) of a number of known myocardial markers in wildtype versus *MZezh2*$^{hu5670/5670}$ embryos at 3.3 hpf.

**Fig. S5. a.** Distribution of percentage of hearts jogging to the left, right, or straight at 1 dpf in *MZezh2*$^{hu5670/+}$ and *MZezh2*$^{hu5670/hu5670}$. Total number of embryos analyzed is depicted at the top of the graph. **b.** *In situ* hybridization for *myl7* (extended staining) and *mef2cb* at 2 dpf in *MZezh2*$^{hu5670/5670}$ and *MZezh2*$^{hu5670/+}$. In *MZezh2*$^{hu5670/+}$ expression is restricted to the heart, whereas in the *MZezh2*$^{hu5670/5670}$ embryos expression is visible in the area surrounding the heart tube. Scale bar is 200 μm. **c.** Whole mount immunostaining for dm-GRASP and active Caspase-3 in *MZezh2*$^{hu5670/5670}$ embryos and heterozygous siblings at 2 dpf. *MZezh2*$^{hu5670/5670}$ embryos show normal expression of dm-GRASP and Caspase-3. The dotted line depicts the

expression of dm-GRASP in the heart. Scale bar is 200 μm. **d.** Immunostaining for GFP *Tg(myl7::GFP)* combined with *in situ* hybridization for *nkx2.5* at 1 and 1.5 dpf in $MZezh2^{hu5670/+}$ and $MZezh2^{hu5670/hu5670}$ embryos. Expression of *nkx2.5* is partially absent in $MZezh2^{hu5670/hu5670}$ embryos at 1.5 dpf compared to $MZezh2^{hu5670/+}$. Scale bar is 50 μm. The numbers indicate the number of embryos with the displayed phenotype compared to the total number of embryos analyzed.

```
Danio rerio     MGLTGRKSEKGPVCWRRRVKSEYMRLRQLKRFRRADEVKSMFSSNRQKILERTD---ILN
Homo sapiens    MGQTGKKSEKGPVCWRKRVKSEYMRLRQLKRFRRADEVKSMFSSNRQKILERTE---ILN
Mus musculus    MGQTGKKSEKGPVCWRKRVKSEYMRLRQLKRFRRADEVKTMFSSNRQKILERTE---TLN
D. melanogaster -----MNSTKVPPEWKRRVKSEYIKIRQQKRYKRADEIKEAWIRNWDEHNHNVQDLYCES
                      :* * *  *::******:::** **::****:*  :   * ::   ...:     .

Danio rerio     QEWKLRRIQPVHIMTPVSSLRGTRECTVDSGFSEFSRQVIPLKTLNAVASVPVMYSWSPL
Homo sapiens    QEWKQRRIQPVHILTSVSSLRGTRECSVTSDL-DFPTQVIPLKTLNAVASVPIMYSWSPL
Mus musculus    QEWKQRRIQPVHIMTSVSSLRGTRECSVTSDL-DFPAQVIPLKTLNAVASVPIMYSWSPL
D. melanogaster KVWQAKPYDPPHVDCVKR-----AEVTSYNGI-PSGPQKVPICVINAVTPIPTMYTWAPT
                : *: :  :* *:           * :   . :      * :*: .:***: :* **:*:*

Danio rerio     QQNFMVEDETVLHNIPYMGDEILDQDGTFIEELIKNYDGKVHGDRECGFINDEIFVELVN
Homo sapiens    QQNFMVEDETVLHNIPYMGDEVLDQDGTFIEELIKNYDGKVHGDRECGFINDEIFVELVN
Mus musculus    QQNFMVEDETVLHNIPYMGDEVLDQDGTFIEELIKNYDGKVHGDRECGFINDEIFVELVN
D. melanogaster QQNFMVEDETVLHNIPYMGDEVLDKDGKFIEELIKNYDGKVHGDKDPSFMDDAIFVELVH
                *********************:**:**.****************:: .*::* ******.

Danio rerio     ALNQYSDNEEDDEEDD-H---------------------HDYKFEKMDLC--DGKDD--
Homo sapiens    ALGQYNDDDDDDDGDD-P---------------------EEREEKQKDLE--DHRDD--
Mus musculus    ALGQYNDDDDDDDGDD-P---------------------DEREEKQKDLE--DNRDD--
D. melanogaster ALMRSYSKELEEAAPGTATAIKTETLAKSKQGEDDGVVDVDADGESPMKLEKTDSKGDLT
                ** :  ..: ::                                  .    .  .*   * : *

Danio rerio     ----------AEDHKEQLSSESHNNDGSKKFPSDKIFEAISSMFPDKGSTEELKEKYKEL
Homo sapiens    ----------------------KESRPPRKFPSDKIFEAISSMFPDKGTAEELKEKYKEL
Mus musculus    ----------------------KETCPPRKFPADKIFEAISSMFPDKGTAEELKEKYKEL
D. melanogaster EVEKKETEEPLETEDADVKPDVEEVKDKLPFPAPIIFQAISANFPDKGTAQELKEKYIEL
                                      .:      **:  **:***: *****:::****** **

Danio rerio     TEQQLPGALPPECTPNIDGPNAKSVQREQSLHSFHTLFCRRCFKYDCFLHPFQA--TPNT
Homo sapiens    TEQQLPGALPPECTPNIDGPNAKSVQREQSLHSFHTLFCRRCFKYDCFLHPFHA--TPNT
Mus musculus    TEQQLPGALPPECTPNIDGPNAKSVQREQSLHSFHTLFCRRCFKYDCFLHPFHA--TPNT
D. melanogaster TEHQDPE-RPQECTPNIDGIKAESVSRERTMHSFHTLFCRRCFKYDCFLHRLQGHAGPNL
                **:* *   * ******** :*:**.**:::****************** ::.   **

Danio rerio     YKRKNMENLVDSKPCGIYCYMYMVQDGMVREYPAGVVPERAKTPSKRSTGRRRGRLPNSN
Homo sapiens    YKRKNTETALDNKPCGPQCYQHLEG---AKEFAAALTAERIKTPPKRPGGRRRGRLPNNS
Mus musculus    YKRKNTETALDNKPCGPQCYQHLEG---AKEFAAALTAERIKTPPKRPGGRRRGRLPNNS
D. melanogaster QKRRYPELKPFAEPCSNSCYMLIDG--MKEKLAAD-----SKTPPIDSCNEASSE-----
                 **:  *     :**.  **  :      .:  *       ***      .  ..

Danio rerio     SRPSTPTVNS-ETKDTDSDREGGA-DGNDSNDKDDDDKKDETTSSSEANSRCQTPVKLKL
Homo sapiens    SRPSTPTINVLESKDTDSDREAGTETGGENNDKEEEEKKDETSSSSEANSRCQTPIKMKP
Mus musculus    SRPSTPTISVLESKDTDSDREAGTETGGENNDKEEEEKKDETSSSSEANSRCQTPIKMKP
D. melanogaster -----------DSND--SNSQF------SNKDFNHENSKDNGLTVNSAAVAEINSIMAGM
                           :::*  *: :       ..:* :.::.**:  : ..*     . :

Danio rerio     SSEPPENVDWSGAEASLFRVLIGTYYDNFCAIARLIGTKTCRQVYEFRVKESSIIARAPA
Homo sapiens    NIEPPENVEWSGAEASMFRVLIGTYYDNFCAIARLIGTKTCRQVYEFRVKESSIIAPAPA
Mus musculus    NIEPPENVEWSGAEASMFRVLIGTYYDNFCAIARLIGTKTCRQVYEFRVKESSIIAPVPT
D. melanogaster MNITSTQCVWTGADQALYRVLHKVYLKNYCAIAHNMLTKTCRQVYEFAQKEDAEFSFEDL
                      :  *:**: :::***  .*  .*:****: : **********  **.: ::

Danio rerio     VDENTPQRKKKRKHRLWATHCRKIQLKKDGSSNHVYNYQPCDHPRQPCDSSCPCVTAQNF
Homo sapiens    EDVDTPPRKKKRKHRLWAAHCRKIQLKKDGSSNHVYNYQPCDHPRQPCDSSCPCVIAQNF
Mus musculus    EDVDTPPRKKKRKHRLWAAHCRKIQLKKDGSSNHVYNYQPCDHPRQPCDSSCPCVIAQNF
D. melanogaster RQDFTPPRKKKKKQRLWSLHCRKIQLKKDSSSNHVYNYTPCDHPGHPCDMNCSCIQTQNF
                 :  ** ****:*:***: **********.******** ***** :*** .* *: :***

Danio rerio     CEKFCQCSSECQNRFPGCRCKAQCNTKQCPCYLAVRECDPDLCLTCGAAEHWDSKNVSCK
Homo sapiens    CEKFCQCSSECQNRFPGCRCKAQCNTKQCPCYLAVRECDPDLCLTCGAADHWDSKNVSCK
Mus musculus    CEKFCQCSSECQNRFPGCRCKAQCNTKQCPCYLAVRECDPDLCLTCGAADHWDSKNVSCK
D. melanogaster CEKFCNCSSDCQNRFPGCRCKAQCNTKQCPCYLAVRECDPDLCQACGAD-QFKLTKITCK
                *****:***:**************************************** :***   ::. .:::**

Danio rerio     NCSIQRGAKKHLLLAPSDVAGWGIFIKEPVQKNEFISEYCGEIISQDEADRRGKVYDKYM
Homo sapiens    NCSIQRGSKKHLLLAPSDVAGWGIFIKDPVQKNEFISEYCGEIISQDEADRRGKVYDKYM
Mus musculus    NCSIQRGSKKHLLLAPSDVAGWGIFIKDPVQKNEFISEYCGEIISQDEADRRGKVYDKYM
D. melanogaster NVCVQRGLHKHLLMAPSDIAGWGIFLKEGAQKNEFISEYCGEIISQDEADRRGKVYDKYM
                *  .:***  :****:****:******:*:  .******************************

Danio rerio     CSFLFNLNNDFVVDATRKGNKIRFANHSVNPNCYAKVMMVNGDHRIGIFAKRAIQTGEEL
Homo sapiens    CSFLFNLNNDFVVDATRKGNKIRFANHSVNPNCYAKVMMVNGDHRIGIFAKRAIQTGEEL
Mus musculus    CSFLFNLNNDFVVDATRKGNKIRFANHSVNPNCYAKVMMVNGDHRIGIFAKRAIQTGEEL
D. melanogaster CSFLFNLNNDFVVDATRKGNKIRFANHSINPNCYAKVMMVTGDHRIGIFAKRAIQPGEEL
                ****************************:***********.************** ****

Danio rerio     FFDYRYSQADALKYVGIEREMEIP
Homo sapiens    FFDYRYSQADALKYVGIEREMEIP
Mus musculus    FFDYRYSQADALKYVGIEREMEIP
D. melanogaster FFDYRYGPTEQLKFVGIEREMEIV
                ******. :: **:*********
```

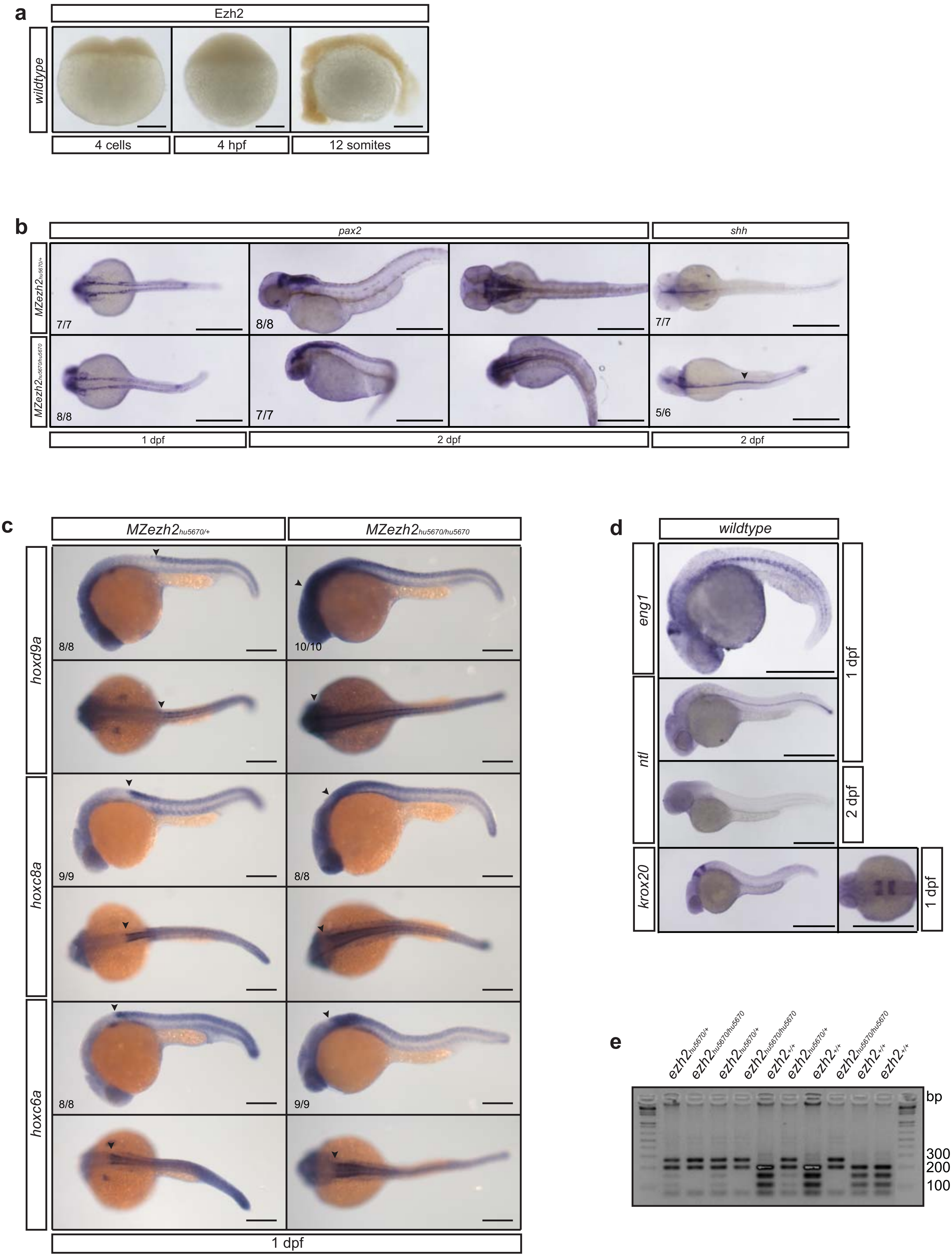

a
Ezh2
wildtype
4 cells
4 hpf
12 somites
b
pax2
shh
MZezh2hu5670/+
MZezh2hu5670/hu5670
7/7
8/8
7/7
8/8
7/7
7/7
5/6
1 dpf
2 dpf
2 dpf
c
MZezh2hu5670/+
MZezh2hu5670/hu5670
hoxd9a
hoxc8a
hoxc6a
8/8
10/10
9/9
8/8
8/8
9/9
1 dpf
d
wildtype
eng1
ntl
krox20
1 dpf
2 dpf
1 dpf
e
ezh2hu5670/+
ezh2hu5670/hu5670
ezh2hu5670/+
ezh2hu5670/hu5670
ezh2+/+
ezh2hu5670/+
ezh2+/+
ezh2hu5670/hu5670
ezh2+/+
ezh2+/+
bp
300
200
100

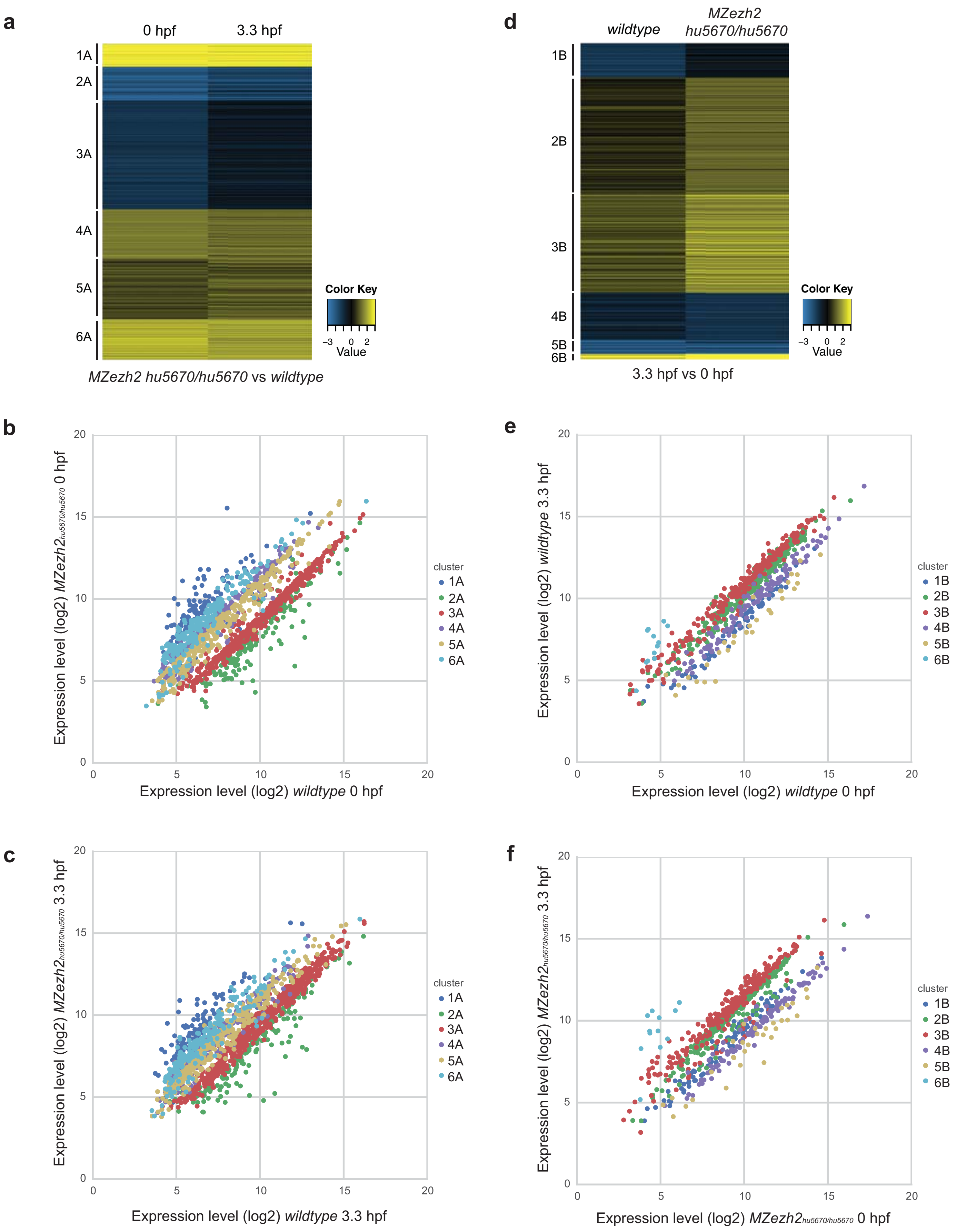
a
0 hpf
3.3 hpf
1A
2A
3A
4A
5A
6A
Color Key
−3 0 2
Value
MZezh2 hu5670/hu5670 vs wildtype
d
wildtype
MZezh2 hu5670/hu5670
1B
2B
3B
4B
5B
6B
Color Key
−3 0 2
Value
3.3 hpf vs 0 hpf
b
Expression level (log2) MZezh2 hu5670/hu5670 0 hpf
Expression level (log2) wildtype 0 hpf
cluster
1A
2A
3A
4A
5A
6A
e
Expression level (log2) wildtype 3.3 hpf
Expression level (log2) wildtype 0 hpf
cluster
1B
2B
3B
4B
5B
6B
c
Expression level (log2) MZezh2 hu5670/hu5670 3.3 hpf
Expression level (log2) wildtype 3.3 hpf
cluster
1A
2A
3A
4A
5A
6A
f
Expression level (log2) MZezh2 hu5670/hu5670 3.3 hpf
Expression level (log2) MZezh2 hu5670/hu5670 0 hpf
cluster
1B
2B
3B
4B
5B
6B

**a**

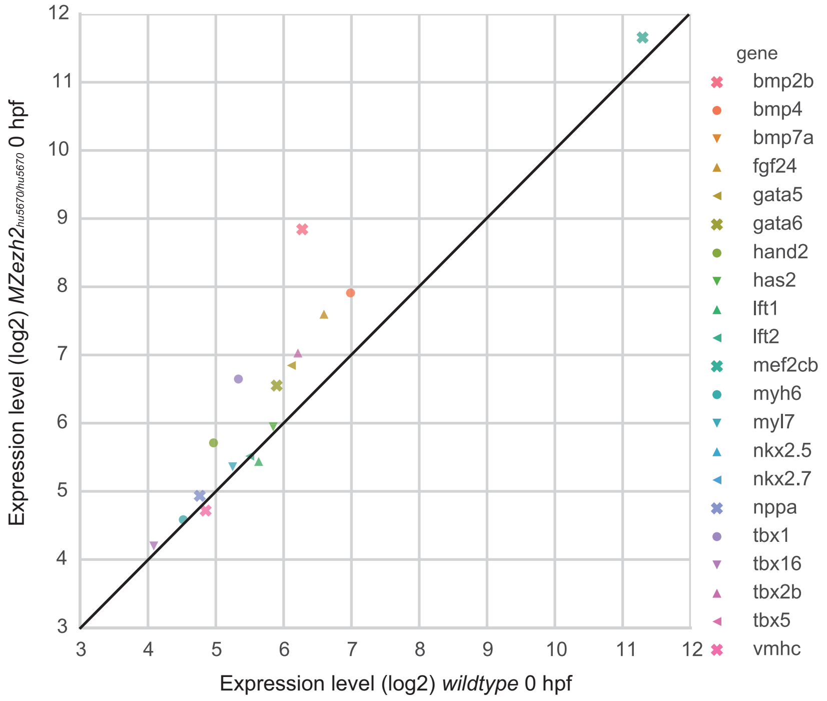
Expression level (log2) *MZezh2*$_{hu5670/hu5670}$ 0 hpf
Expression level (log2) *wildtype* 0 hpf
3 4 5 6 7 8 9 10 11 12
gene
bmp2b
bmp4
bmp7a
fgf24
gata5
gata6
hand2
has2
lft1
lft2
mef2cb
myh6
myl7
nkx2.5
nkx2.7
nppa
tbx1
tbx16
tbx2b
tbx5
vmhc

**b**

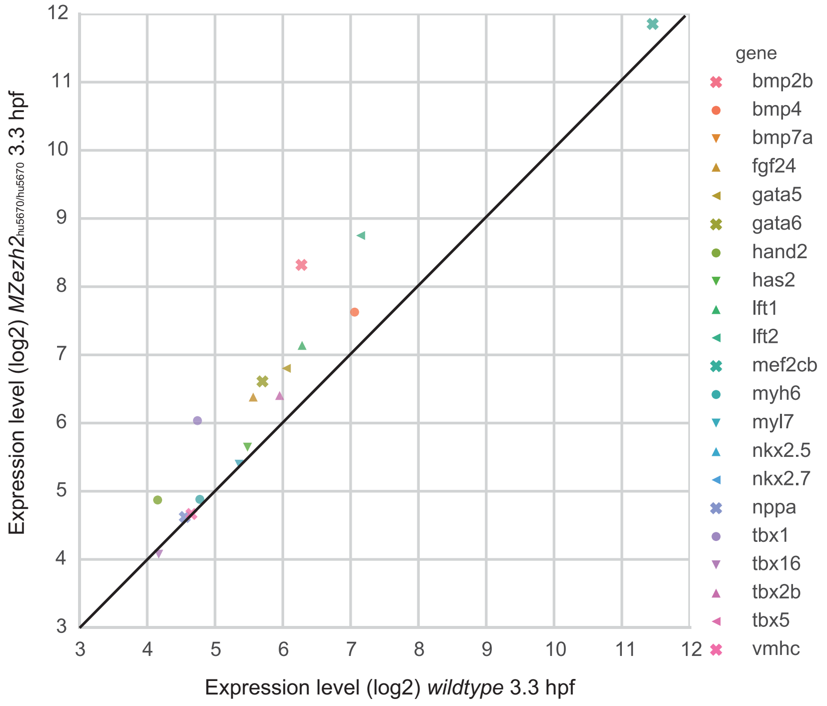
Expression level (log2) *MZezh2*$_{hu5670/hu5670}$ 3.3 hpf
Expression level (log2) *wildtype* 3.3 hpf
3 4 5 6 7 8 9 10 11 12
gene
bmp2b
bmp4
bmp7a
fgf24
gata5
gata6
hand2
has2
lft1
lft2
mef2cb
myh6
myl7
nkx2.5
nkx2.7
nppa
tbx1
tbx16
tbx2b
tbx5
vmhc

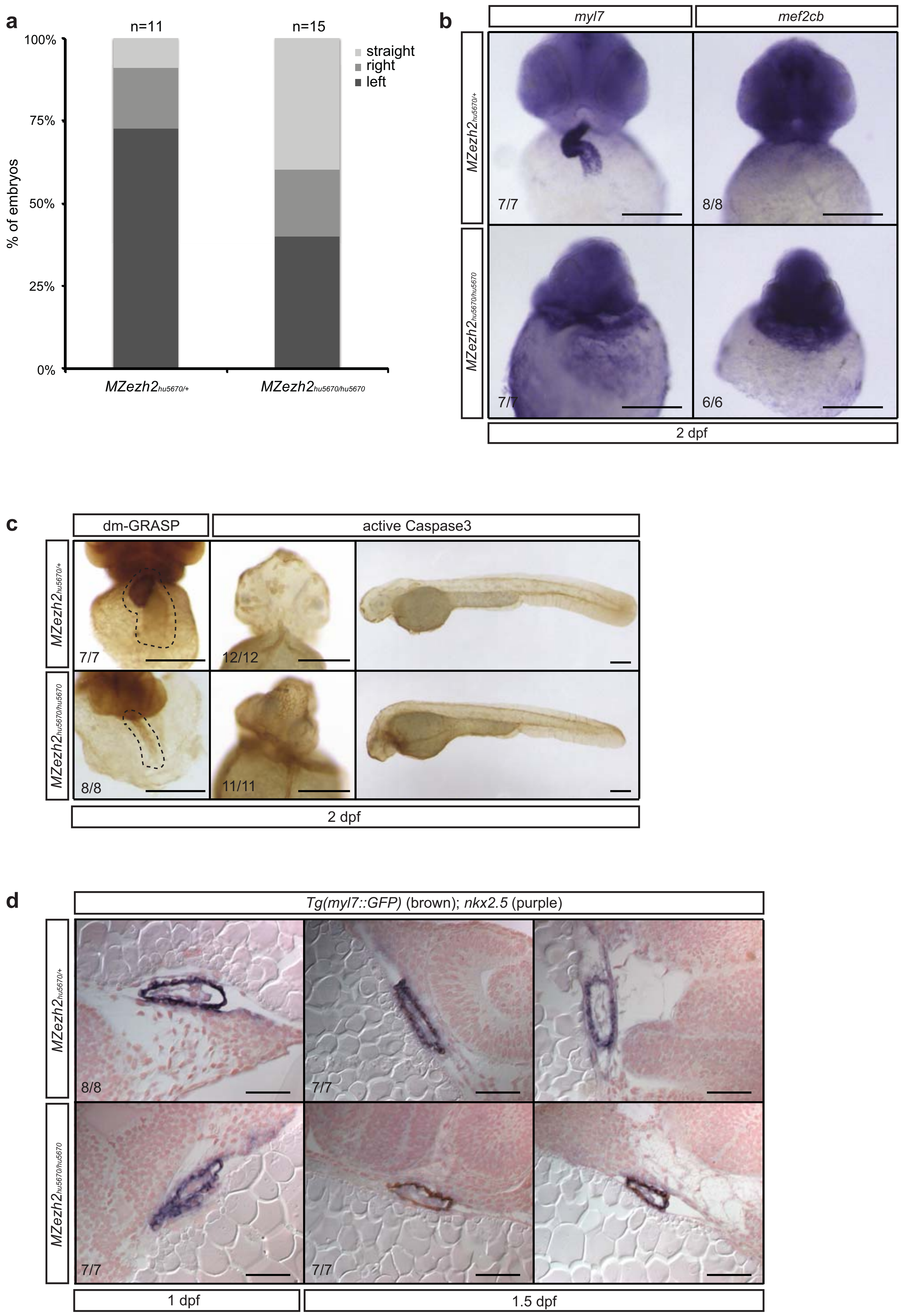
a
n=11
n=15
100%
75%
50%
25%
0%
% of embryos
straight
right
left
MZezh2hu5670/+
MZezh2hu5670/hu5670
b
myl7
mef2cb
MZezh2hu5670/+
MZezh2hu5670/hu5670
7/7
8/8
7/7
6/6
2 dpf
c
dm-GRASP
active Caspase3
MZezh2hu5670/+
MZezh2hu5670/hu5670
7/7
12/12
8/8
11/11
2 dpf
d
Tg(myl7::GFP) (brown); nkx2.5 (purple)
MZezh2hu5670/+
MZezh2hu5670/hu5670
8/8
7/7
7/7
7/7
1 dpf
1.5 dpf

SUPPLEMENTARY TABLES

**Table S1.** GO terms list of genes in clusters Fig. 4c. Differentially expressed genes between *MZezh2*$^{hu5670/hu5670}$ versus wildtype embryos at 0 hpf and 3.3 hpf.

**Table S2.** GO terms list of genes in clusters Fig. 4g. Differentially expressed genes between 3.3 hpf versus 0 hpf in *MZezh2*$^{hu5670/hu5670}$ and wildtype embryos.

**Table S3.** Primers used for genotyping *ezh2(hu5670)*. First PCR is performed with primer pair 1 and 4, followed by a nested PCR with primer pair 2 and 3.

**Table S4.** Primers used for qPCR.

**Supplementary Table S1.** List of genes in clusters Figure 4b,c for which significantly enriched biological functions were identified by DAVID analysis. Differentially expressed between *MZezh2* mutant versus wildtype embryos at 0 hpf and 3.3hpf.

| **Cluster 1** | **Cluster 2** | **Cluster 3** | **Cluster 4** | **Cluster 5** | **Cluster 6** |
|---|---|---|---|---|---|
| *dlx4b* | - | *adat1* | - | *aplnrb* | *cxcr4b* |
| *esrp1* | | *cct3* | | *ccnd1* | *cyp26a1* |
| *her8a* | | *cct6a* | | *cdh2* | *emx3* |
| *hnf1bb* | | *e2f4* | | *celf2* | *irx7* |
| *hoxa13a* | | *exosc5* | | *clstn1* | *lama1* |
| *hoxd13a* | | *fars2* | | *dlx4a* | *nr4a2b* |
| *ivns1abpa* | | *fkbp11* | | *dlx6a* | *otpb* |
| *nkx6.2* | | *fkbp3* | | *dmrt2b* | *sema3ab* |
| *pax6b* | | *fkbp7* | | *eaf2* | *sema3d* |
| *prrx1a* | | *med11* | | *ehmt1a* | *smo* |
| *rassf8b* | | *med19a* | | *epdr1* | *sox3* |
| *smad7* | | *med4* | | *esrrgb* | *vegfaa* |
| *tbx4* | | *mrpl41* | | *fbln5* | |
| *tfap2a* | | *mybbp1a* | | *fgf24* | |
| *tsku* | | *nhp2* | | *figla* | |
| *vsx1* | | *nip7* | | *foxd5* | |
| | | *nop16* | | *foxi1* | |
| | | *pin1* | | *fstl1b* | |
| | | *piwil2* | | *her11* | |
| | | *pno1* | | *her2* | |
| | | *polr2gl* | | *hsp70l* | |
| | | *ppwd1* | | *igfbp2a* | |
| | | *rpia* | | *invs* | |
| | | *scnm1* | | *irx1b* | |
| | | *taf1a* | | *lbr* | |
| | | *top1mt* | | *lbx1a* | |
| | | *tpi1a* | | *lbx2* | |
| | | *utp15* | | *mak16* | |
| | | *vbp1* | | *mmp14a* | |
| | | *wdr55* | | *mmp14b* | |
| | | *zgc:113019* | | *mxtx2* | |
| | | *zgc:163098* | | *nav3* | |
| | | *zmat5* | | *nfia* | |
| | | | | *nr2f1b* | |
| | | | | *pax6a* | |
| | | | | *pax9* | |
| | | | | *plcd1b* | |
| | | | | *psmb11* | |
| | | | | *shox2* | |
| | | | | *smad5* | |
| | | | | *sox19a* | |
| | | | | *tcea2* | |
| | | | | *ufm1* | |
| | | | | *unc5b* | |
| | | | | *vax1* | |
| | | | | *vox* | |
| | | | | *zgc:158291* | |

**Supplementary Table S2.** List of genes in clusters Figure 4f,g for which significantly enriched biological functions were identified by DAVID analysis. Differentially expressed between 3.3 hpf versus 0 hpf in *MZezh2* mutant and wildtype embryos.

| Cluster 1 | Cluster 2 | Cluster 3 | Cluster 4 | Cluster 5 | Cluster 6 |
|---|---|---|---|---|---|
| - | - | *cbx8a* | - | - | *foxd5* |
| | | *cep63* | | | *her5* |
| | | *dctn2* | | | *irx7* |
| | | *exosc4* | | | *mxtx2* |
| | | *med19a* | | | *vox* |
| | | *mybbp1a* | | | |
| | | *nip7* | | | |
| | | *nop16* | | | |
| | | *pes* | | | |
| | | *pno1* | | | |
| | | *rfc2* | | | |
| | | *sept4a* | | | |
| | | *sox19a* | | | |
| | | *suv420h1* | | | |
| | | *taf1a* | | | |
| | | *top1mt* | | | |
| | | *utp15* | | | |
| | | *wdr55* | | | |

**Supplementary Table S3.** Primers to genotype *ezh2(hu5670).*

| | |
|---|---|
| Ezh2_14-15_1 | AAAGTTGATTGACCCAGCTC |
| Ezh2_14-15_4 | TGATACTGAGCTTTGTTTGC |
| Ezh2_14-15_2 | TGTAAAACGACGGCCAGTAAACCTCCTTACTGAAACAGG |
| Ezh2_14-15_3 | AGGAAACAGCTATGACCATTGACAATTGTATGACTCACAGG |

Primers are depicted 5' to 3' end. A touch down PCR was performed with primers 1 and 4, followed by a PCR with primers 2 and 3. Subsequently the PCR product was digested with RsaI to complete genotyping. e*zh2(hu5670)* results in a loss of a RsaI site.

**Supplementary Table S4.** Primers used for qPCR.

| | |
|---|---|
| ezh1-198_Fw | AGGAAGCGTCTAGTGAGGTCT |
| ezh1_381_Rv | ACGGCGATTTGACTGGAACA |
| ezh2_Fw | AAATCGGAGAAGGGTCCTGT |
| ezh2_Rv | TCTGTTGGAGCTGAACATGC |
| ef1a_Fw | TTGAGAAGAAAATCGGTGGTGCTG |
| ef1a_Rv | GGAACGGTGTGATTGAGGGAAATTC |
| b-actin_Fw | CGAGCAGGAGATGGGAAC |
| b-actin_Rv | CAACGGAAACGCTCATTGC |

Primers are depicted 5' to 3'.

SUPPLEMENTARY MOVIES

**Movie S1.** Time lapse imaging between 1 and 2 dpf of heterozygous siblings (*MZezh2*$^{hu5670/+}$) in *Tg(myl7::GFP)* background.

**Movie S2.** Time lapse imaging between 1 and 2 dpf of *MZezh2* mutants in *Tg(myl7::GFP)* background.
